# *Leishmania* exosomal tRNA-derived small non-coding RNA fragments modulate host THP-1 derived macrophage proteins: A quantitative proteomic analysis

**DOI:** 10.1371/journal.pone.0356144

**Published:** 2026-08-19

**Authors:** Harsimran Kaur Brar, Atieh Moradimotlagh, Dilraj Kaur Longowal, Kyung-Mee Moon, Leonard J. Foster, Alice L-F Mui, Neil Reiner, Devki Nandan

**Affiliations:** 1 Division of Infectious Diseases, Department of Medicine, University of British Columbia, Vancouver, British Columbia, Canada; 2 Department of Biochemistry and Molecular Biology, University of British Columbia, Vancouver, British Columbia, Canada; 3 Department of Surgery, University of British Columbia, Vancouver, British Columbia, Canada; Zoological Survey of India, INDIA

## Abstract

Protozoan parasites of the genus *Leishmania,* the causative agents of human leishmaniasis, have evolved mechanisms to manipulate host cell functions to their advantage. Recently, small non-coding RNAs have been identified as contributors to the pathogenesis of *Leishmania* infection. An increasing number of studies have demonstrated that *Leishmania* tRNA-derived small non-coding RNAs, also known as *Leishmania* tRNA-derived fragments (Ld-tRFs), can be delivered to host cells via exosomes, thereby influencing host cell function. Here, we investigated the potential effects of *Leishmania* exosomal Ld-tRF-Asp and Ld-tRF-Leu in THP-1-derived host macrophages. SILAC (Stable Isotope Labelling by Amino acids in Cell culture) based quantitative proteomics was used to investigate the effects of synthetic Ld-tRF-Asp and Ld-tRF-Leu by analyzing how these Ld-tRFs affect the macrophage proteome. Ld-tRF-Asp modulated 20 proteins, whereas Ld-tRF-Leu affected 18 proteins in macrophages. Interestingly, 7 of the Ld-tRF-Asp-modulated proteins and 7 of the Ld-tRF-Leu-modulated proteins showed potential pro-*Leishmania* effects. Biochemical isolation of Argonaute (Ago) protein complexes by “Ago proteins Affinity Purification by Peptide,” followed by identification of interacting small non-coding RNAs, revealed the selective presence of both tRFs in *Leishmania-*infected macrophages. This striking finding suggested that Ld-tRFs engage macrophage Ago proteins for their effects. In addition, an *in vivo* biotin-RNA pull-down assay showed that Ld-tRF-Leu selectively interacted with Ago 1, suggesting that Ago 1 is the preferred Ago guiding Ld-tRFs to target host genes. Taken together, this study shows, for the first time, that *Leishmania* exosomal tRFs significantly alter the host macrophage proteome in favour of *Leishmania* survival. This may offer new insights into the mechanisms of leishmaniasis and may provide future therapeutic interventions targeting these regulatory molecules.

## Introduction

Leishmaniasis is caused by an obligate intracellular protozoan parasite from the genus *Leishmania.* Despite significant advances in research, the disease remains prevalent among resource-limited populations across more than 90 countries. Its main clinical forms are cutaneous, mucosal, and visceral leishmaniasis. *L. donovani* is the main causative agent of visceral leishmaniasis, which could be fatal if left untreated. Risk factors include poverty, malnutrition, armed conflicts, tourism, and climate change. Treatment challenges involve high drug toxicity, cost, and increasing drug resistance [1,2]. Although leishmaniasis is a complex disease, a thorough understanding of host-*Leishmania* interactions is essential for developing effective therapies.

Non-coding RNAs (ncRNAs) are emerging players in host-pathogen interactions [3]; however, their exact roles and associated molecular mechanisms remain unclear. *Leishmania*, an intracellular pathogen, has evolved mechanisms that enable it to adapt to and survive within the host’s hostile environment. One of the primary strategies used by *Leishmania* to modify host responses in favour of its survival involves remodelling the host transcriptome, including the regulation of both protein-coding RNAs and ncRNAs, such as microRNAs (miRNAs) [4–7].

In addition to directly modulating host macrophage ncRNAs, *Leishmania* can also deliver its own ncRNAs (mainly transfer RNA -tRNA-, and ribosomal RNA -rRNA- fragments) via secretory exosomes, thereby influencing the host environment. Previously, our group and others have shown that during *Leishmania* infection, secretory exosomes can modify the host immune response [8–10]. These vesicles can impact immune responses in various ways; for example, the parasite releases exosomes in sand flies and host cells, which can trigger inflammation and exacerbate cutaneous leishmaniasis [11]. In another related study, our group demonstrated that pre-treating mice with *L. donovani* exosomes prior to infection worsens the disease by increasing IL-10 and decreasing TNF-α production [8]. Overall, *Leishmania* exosomes that carry ncRNAs, such as tRNA and ribosomal RNA fragments, mainly appear to suppress the immune response and play a critical role in shifting the host’s immune system to promote parasite survival. Transfer RNA fragments (tRFs) and other small RNAs, such as ribosomal RNA fragments found in *Leishmania’s* exosomes, suggest roles in intercellular communication and potentially host translation regulation [12,13]. While progress has been made in understanding how *Leishmania* alters host ncRNAs, particularly miRNAs, how parasite exosomal ncRNAs, especially tRFs, influence host cell biology remains unknown.

Transfer RNAs (tRNA) are small noncoding RNAs (70–90 nucleotides) that play an established role in protein synthesis by transporting amino acids to the ribosome [14]. It is becoming clear that tRNAs can be precisely cleaved by specific enzymes into multiple small RNA fragments, generating different classes of tRNA-derived RNA fragments, such as 5′tRFs, 3′tRFs, and tRNA halves [15]. These non-random fragments are collectively referred to as tRFs and have been implicated in non-canonical roles in biological functions such as mRNA expression silencing, regulation of cellular stress responses, DNA damage repair, and regulation of cell proliferation/differentiation [14]. Interestingly, tRFs have also been shown to play a role in parasite biology [10]. In several infections—such as those caused by Trichomonas, Trypanosoma, HCV, and HBV—tRFs have been detected within extracellular vesicles (EVs) secreted by the pathogens. These EV-delivered tRFs are transferred to host cells, where they can modulate host gene expression via cross-kingdom RNAi mechanisms [16,17].

This study examined the potential effect of *Leishmania* exosomal tRFs on host cell biology. We selected the two most abundant tRFs from *L. donovan*i secretory tRFs (Ld-tRF-Asp and Ld-tRF-Leu) based on our previous study [12]. PMA-differentiated THP-1 cells were transfected with tRF-Leu or tRF-Asp mimics, followed by quantitative proteomic analysis by mass spectrometry to identify changes in the host proteome. Out of the total 1736 host proteins unambiguously identified, 38 proteins were significantly modulated by Ld-tRFs. Interestingly, 14 of these proteins were found to be relevant to support the pro-parasitic environment. Furthermore, we show that both *Leishmania* tRFs preferentially load onto host Ago 1, potentially influencing host gene expression, suggesting a possible mechanism of action.

## Materials and methods

### Reagents and antibodies

THP-1 (TIB-202TM), the human monocytic cell line, was obtained from the American Type Culture Collection (ATCC). RPMI 1640 medium and fetal bovine serum (FBS) were purchased from Gibco. L-glutamine, HEPES, Hanks’ balanced salt solution (HBSS), penicillin/streptomycin, M199 medium, hemin, folic acid, adenosine, and phorbol 12-myristate 13-acetate (PMA), along with Amicon® Ultra-2 centrifugal filter devices (UFC200324), were acquired from Millipore Sigma. Anti-Lamin A/C (#2032), anti-Ago1 (#5053), anti-Ago2 (#2897), and anti-Ago3 (#5054) were purchased from Cell Signaling. Anti-actin (SC-47778) was obtained from Santa Cruz Biotechnology. Anti-GAPDH (G041) was acquired from Applied Biological Materials Inc. The GeneJET RNA Purification kit (K0732), North2South Hybridization buffer (#37549), North2South Hybridization Stringency Wash Buffer (#37555), and Chemiluminescent Nucleic Acid Detection Module kit (#89880) were purchased from Thermo Scientific. Stable isotope-labeled amino acids were purchased from Cambridge Isotope Laboratories (Andover, MA, USA). *Leishmania* exosomal tRFs (tRF-Asp TTCTCGGTAGTATAGTGGTTAGTATACCCGCC, tRF-Asp-Scrambled GCTAATTCGTCTATACTCGGTGCGCTGTGAAT tRF-Leu GGTGAGATGGTCGAGTGGTCT and tRF-Leu-scrambled AGGTTGCGATGGACGGTTGTG) were obtained from Dharmacon, now IDT. Sequences of tRF-Asp and tRF-Leu were based on Ulrike et al [12].

### THP-1 cell culture, stable isotope labeling by amino acid in cell culture (SILAC) labeling and differentiation

THP-1 cells were cultured at 37°C with 5% CO2 in RPMI-1640 media containing 10% heat-inactivated fetal calf serum (FBS), 10 mM HEPES, 100 units/mL penicillin-streptomycin, and 2 mM L-glutamine (Gibco). For SILAC, THP-1 cells were grown in L-glutamine and L-arginine and L-lysine-deficient RPMI-1640 (Caisson Labs), supplemented with 100 units/mL penicillin-streptomycin, 2 mM L-glutamine, 7% dialyzed fetal bovine serum and labeled with either L-arginine-^13^C_6_, L-lysine ^2^H_4_ (Arg6, Lys4) or L-arginine ^13^C_6_-^15^N_4_ and ^13^C_6_,^15^N_2_-lysine (Lys8, Arg10) as described [18,19]. For experiments, labeled THP-1 cells were differentiated with 10 ng/mL of PMA for 16–18 h. The differentiated THP-1 cells (dTHP-1) were rested in fresh medium for 24 h before Ld-tRFs were delivered by transfection.

### *Leishmania donovani* culture and infection of dTHP-1 cells

This study utilized the Sudan S2 strain of *Leishmania*, obtained from K.P. Chang. Promastigotes were cultured with passages every 3 days for 15–20 passages. To maintain the strain’s virulence and infectious capacity, fresh amastigotes were isolated from the spleens of infected Syrian Golden hamsters, then transformed into promastigotes in vitro and cultured as described previously [20]. Briefly, parasites were grown in M199 medium supplemented with 10% heat-inactivated FBS, 20 mM HEPES, 10 μg/mL folic acid, 3 μg/mL hemin, 2 mM L-glutamine, 100 U/mL penicillin/streptomycin, and 100 μM adenosine at 26°C. For *in vitro* infection, dTHP-1 cells were incubated with stationary-phase promastigotes at an MOI (*Leishmania*: THP-1) of 20:1. The infection rate was calculated as previously described [19]. The infection rate ranged from 60 to 80%. For most of the experiments, infection rates were determined in triplicate.

### Liquid chromatography-tandem mass spectrometry (LC/MS-MS) and protein identification

Cell pellets from control and transfected cells from three independent experiments were boiled in 10% SDS, 100 mM Tris, pH 8.8. Protein concentrations were estimated by BCA Protein Assay Kit (Thermo Scientific). Approximately 20 μg of each of 3-plex SILAC labelled set within the same experiment were pooled, and proteins were precipitated by incubating with 80% cold acetone at −20°C overnight. Resulting protein pellets were washed with cold 80% acetone and re-solubilized with 6M urea, 2M thiourea, 100 mM Tris pH 8.0. The resulting protein solutions were digested in a multi-step overnight process with MS-grade LysC and Trypsin [21,22]. Lys-C cleavage specificity overlaps with trypsin. Trypsin cleaves at the C-terminal ends of Arginine and lysine; Lys-C cleaves at the C-terminus of lysine. However, trypsin cleaves lysine less efficiently than arginine; therefore, Lys-C was added to compensate for this difference [23]. The resulting peptides were cleaned on C-18 STop And Go Extraction (STAGE) Tips [24]. One-tenth (2 μg) of the total cleaned peptides were loaded onto a quadrupole–time of flight mass spectrometer (Impact II; Bruker Daltonics) coupled to an Easy nano LC 1000 HPLC (Thermo Fisher Scientific) using a 40−50 cm long analytical column packed with 1.9μm-diameter Reprosil-Pur C-18-AQ beads (Dr. Maisch, www.Dr-Maisch.com), fixed on an in-house constructed column heater set at 50°C. Buffer A consisted of 0.1% formic acid in water, and buffer B consisted of 0.1% formic acid and 80% acetonitrile in water. Samples were resuspended in buffer A and analyzed over a 180-minute separation. The column was then washed with 100% Buffer B for 15 min, and re-equilibrated with Buffer A. The analysis was performed at 0.25μl/min flow rate and the instrument was set in a data-dependent auto-MS/MS mode with inactive focus fragmenting the 20 most abundant ions (one at the time at 18 Hz rate) after each full-range scan from m/z 200Th to m/z 2000Th (at 5 Hz rate). The isolation window for MS/MS was 2–3Th, depending on parent ion mass to charge ratio and the collision energy ranged from 23 to 65eV, depending on ion mass and charge. Parent ions were then excluded from MS/MS for the next 0.4 min and reconsidered if their intensity increased more than 5 times. Singly charged ions were excluded. The error of mass measurement is maintained to be less than 10 ppm. The nano ESI source was operated at 1700V capillary voltage, 0.20 Bar nano buster pressure, 3L/min drying gas and 150°C drying temperature.

Acquired data were searched with MaxQuant version 1.5.1.0 [25] against Uniprot’s human sequence using reverse decoy mode. Heavy labels of arginine and lysine (K4, R6, K8, and R10) were set for quantitation, enabling the re-quantify option. Other parameters were as follows: peptide mass accuracy 10 ppm; fragment mass accuracy 0.05Da; trypsin enzyme specificity, carbamidomethyl as fixed modification, methionine oxidation and N-acetyl protein as variable modifications. The false rate discovery rate was set at 1% at both protein and PSM (Peptide spectrum match) levels. The mass spectrometry proteomics data have been deposited to the ProteomeXchange Consortium via the PRIDE [26] partner repository with the dataset identifier PXD072695 and Token: iKMGjgVSEDFg.

### Quantitative and statistical analysis of liquid chromatography-tandem mass spectrometry data

All downstream analysis of the MaxQuant normalized ratios was done using R software (http://www.r-project.org). Of the total proteins detected by LC-MS/MS, we removed contaminants by filtering against the CRAPome database [27]. Proteins were further filtered to retain only those with at least two unique peptides per protein identification, identified in at least two of the three replicates, with variability less than 30%. The one-sample T-test was performed to statistically compare normalized ratios to 1 (meaning no change). A p-value of less than 0.1 was considered statistically significant. Proteins were considered significantly modulated if their average normalized ratio differed by at least 20% from 1, with p < 0.1. A cut-off was set at 20% from 1 as the minimum change for a protein to be considered modulated.

### Delivery of Ld-tRFs mimics into dTHP-1 cells

Based on the previous work by our lab [18,19], synthetic RNA Ld-tRF-Asp, Ld-tRF-Leu oligos, and scrambled controls (Integrated DNA Technologies) were transfected into dTHP-1 cells (1 × 10^6 cells/ 60 mm plate/2 ml media) at 50 nM using HiPerfect reagent (Invitrogen) for 24 h for a SILAC-related proteomic study. For RNA pull-down assays, a 3′-UTR biotinylated Ld-tRF-Leu oligo and the corresponding scrambled control oligo at 50 nM with HiPerfect reagent were used to transfect THP-1 cells for 24 h.

### Subcellular fractionation

Subcellular fractionation was performed as previously described, with minor modifications [19,28]. Briefly, cells were lysed in hypotonic lysis buffer (HLB) (10 mM Tris-HCl, pH 7.5, 10 mM NaCl, 3 mM MgCl2, 0.3% NP-40, 1 mM NaF, 1 mM Na3VO4) supplemented with protease inhibitors, then passed through a 22-gauge needle to disrupt cells. The lysate was centrifuged at 2,300 × g, and the resulting supernatant was collected as the cytoplasmic fraction. The remaining nuclear pellet was washed, heated at 95°C in 2X SDS-PAGE sample loading buffer, and subjected to Western blotting. Proteins extracted from each fraction were analysed by Western blot using an anti-GAPDH antibody as a cytosolic marker.

### Biotin-RNA pull-down assay

To assess a possible association of Ld-tRF with endogenous Ago proteins, synthetic 3′-UTR biotinylated Ld-tRF-Leu mimic (50 nM) and the corresponding scrambled control oligo (50 nM) were delivered into dTHP-1 cells by transfection. After 24 h of oligo treatment, the RNA pull-down assay was performed. Briefly, 1 × 10^6^ cells were used for each assay. Whole-cell lysates were incubated with streptavidin magnetic beads overnight at 4 °C on an orbital shaker to pull down Ld-tRF-Leu-interacting proteins. RNAse inhibitor (RNaseOUT, final concentration of 0.4 U/μl from Invitrogen) was added to prevent RNA degradation. RNA complexes were collected on paramagnetic streptavidin-conjugated Dynabeads, washed, and the bound material was analyzed by Western blotting for the presence of Ago proteins (Ago1, Ago2, and Ago3). An aliquot from each sample was saved as input material for Western blot analysis.

### Preparation of GST and GST-T6B affinity beads

Ago protein Affinity Purification by Peptides (Ago-APP) was performed as previously described [19,29]. Briefly, to prepare the affinity beads, glutathione-agarose beads (GE-HealthCare) were incubated with GST (control) or GST-T6B recombinant proteins for at least 3 h. The cytoplasmic fractions were prepared in HLB containing EDTA (5 mM) and DTT (0.5 mM).

### GST-T6B pull-down assay for interacting sncRNAs from control and *Leishmania*-infected cells

Cytoplasmic fractions from control and infected cells were used to isolate Ago-interacting RNA using GST-T6B beads, as described previously [29] with some modifications. Briefly, after Ago-APP, 10% of the glutathione-agarose beads were used for Western blotting of Ago1 and Ago2 to confirm the functionality of Ago-APP. The remaining 90% of the beads were used for RNA isolation. First, the beads were incubated with 200 μl of Proteinase K buffer (200 mM Tris-HCl, pH 7.5, 300 mM NaCl, 25 mM EDTA, 2% (w/v) SDS) containing 0.16 mg/ml proteinase K for 15 min at 65°C. Next, the beads were removed by centrifugation at 2,650 × g for 2 min. 200 μl of Phenol:Chloroform:Isoamyl alcohol (25:24:1) was then added to the resulting supernatant, mixed vigorously, and incubated at room temperature (RT) for 15 min, followed by centrifugation at 15,300 × g for 15 min. An equal volume of isopropanol and 30 μg/ml glycogen (Ambion) were added to the aqueous layer containing RNA and incubated overnight at −20°C to facilitate RNA precipitation. The resulting RNA pellet was washed with cold 70% ethanol, air-dried briefly, and then dissolved in DEPC-treated water.

### Small RNA isolation from total RNA

Total RNA was first isolated from uninfected and *Leishmania* infected cells using the GeneJET RNA Purification kit, following the manufacturer’s instructions. After loading the isopropanol-containing lysate onto the column and centrifuging, RNA species larger than 200 bp bound to the column matrix, while RNA smaller than 200 bp passed through in the flow-through. To enrich for small RNAs, the flow-through fraction was collected, and 30 µg/ml glycogen was added. The mixture was incubated overnight at –20 °C to precipitate small RNAs. The resulting RNA pellet was washed with cold 70% ethanol, briefly air-dried, and resuspended in DEPC-treated water.

### Northern blotting

Northern blotting was performed on the GST-T6B pulled-down RNAs as described before [18,30]. Briefly, to the isolated RNA, an equal volume of 2X RNA loading dye (5 mM EDTA, 0.01% bromophenol blue and 95% formamide) was added, followed by heating at 70°C for 5 min and snap-cooling on ice as described. RNA samples were run on a 15% denaturing polyacrylamide TBE/7M urea gel. The gel was pre-run in TBE at 100 V for at least 10 min [31]. Between 0.9 and 3 μg of RNA was loaded and subjected to electrophoresis. RNA was then transferred onto a positively charged Hybond N+ nylon membrane (Amersham Biosciences) at 10 V for 90 min at 4°C. The membrane was UV-cross-linked using a Stratalinker 2400 (Stratagene) and dried at 50°C for 45 min. Pre-hybridization involved incubating the membrane in North2South Hybridization buffer on a shaker at 55°C for 30 min. For overnight hybridization (18–20 h), biotinylated probes for Ld-tRF-leu, Ld-tRF-Asp, and let7a-5p miRNA were added at 50 ng/ml to the pre-hybridization buffer. The let7a-5p probe (5′-/Biosg/AACTATACAACCTACTACCTCA-3’) was synthesized by IDT based on a published report [29]. The Ld-tRF-Leu (5′-/Biosg/AGACCACTCGACCATCTCA −3’) and Ld-tRF-Asp (5′-/Biosg/GGCGGGTATACTAACCACTATAC-3’) probes, also by IDT, were obtained based on the probe used in the previous study [12]. These probes target an internal region of the tRNA sequence to detect RNA species containing this RNA body, including full-length tRNA and tRNA-derived fragments.

After hybridization, the blot was washed three times for 15 min each with diluted North2South Hybridization Stringency Wash Buffer (1X) at 55°C with agitation. The subsequent steps were carried out according to the manufacturer’s instructions for the Chemiluminescent Nucleic Acid Detection Module kit. The membrane was blocked in a nucleic acid detection blocking buffer for 15 min with gentle shaking at room temperature. It was then incubated with Streptavidin: HRP conjugate (1:300) in the same buffer for 15 min at room temperature with agitation. The membrane was washed four times for 5 min each with 1X wash buffer at room temperature with gentle shaking, then incubated with Substrate Equilibration Buffer for 5 min at room temperature. Finally, the membrane was exposed to the working Enhanced Chemiluminescence solution and then to Medical Blue X-ray film for signal detection.

## Results

### Identification of *Leishmania*-tRNA fragments Leucine (Ld-tRF-Leu) or Aspartate (Ld-tRF-Asp) modulated macrophage proteins through global proteomic quantification

Our previous work showed that *Leishmania*-derived exosomes are enriched in a conserved set of small non-coding RNAs (sncRNAs), particularly fragments originating from rRNAs and tRNAs, and that these vesicles efficiently deliver their RNA cargo to host macrophages [12]. Based on these observations, we set out to investigate the biological relevance of tRFs previously identified in exosomes [12], on the macrophage phenotype. We synthesized mimics of the two most abundant tRFs identified in our previous study [12].

Our method for thoroughly identifying and analyzing host macrophage proteins affected by *Leishmania* tRFs involved transfecting macrophages with either a tRF control oligo (scrambled oligos) or *Leishmania* tRFs. This was followed by SILAC-based quantitative LC-MS/MS analysis (see S1 Fig.). After 24 hours of incubation with the respective tRFs, the cells were lysed, and proteins were identified and quantified using liquid chromatography-tandem mass spectrometry (LC-MS/MS), as detailed in the “Materials and Methods” section.

**Fig 1:**
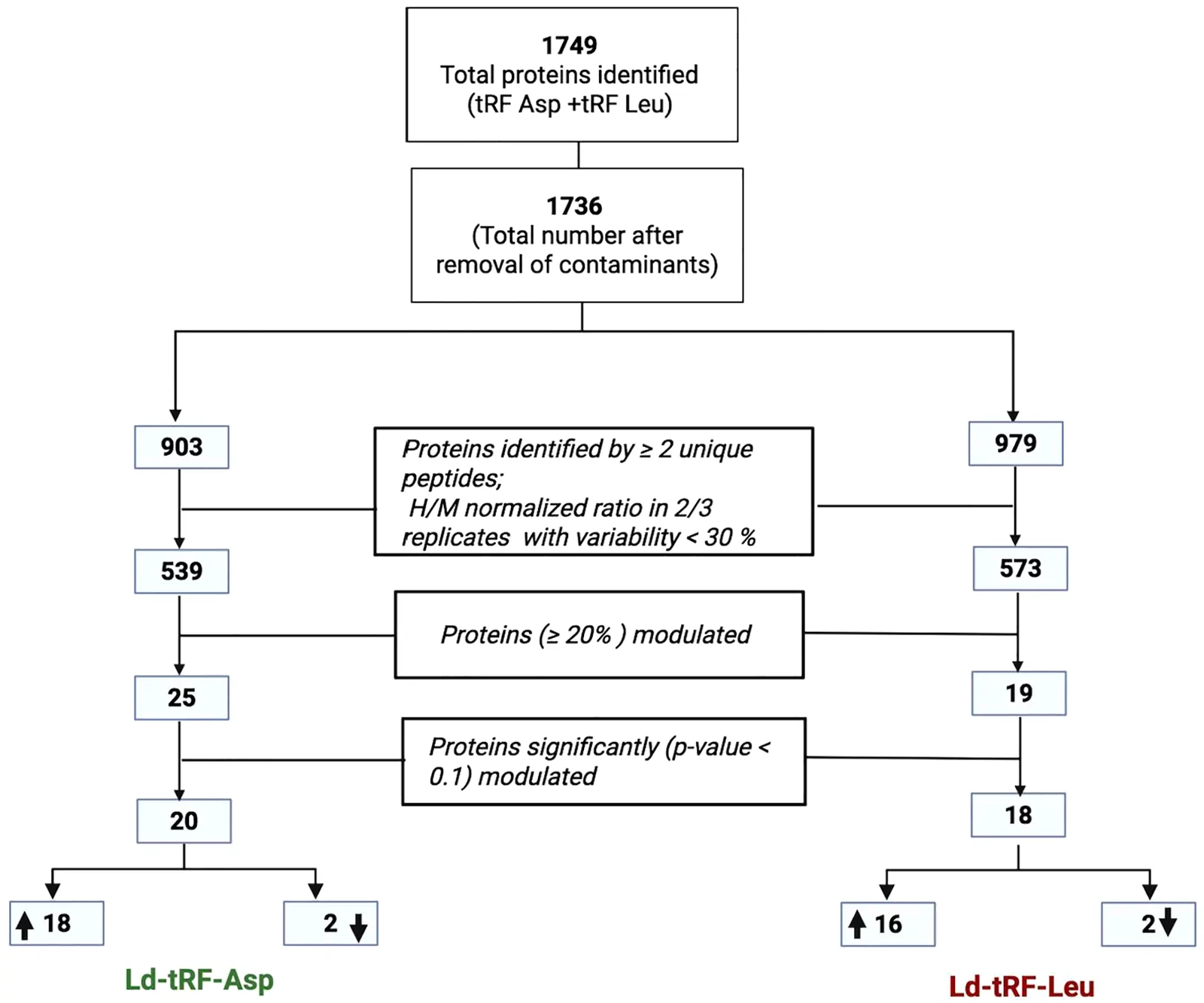
Quantitative proteomic analysis of macrophage proteins upon exposure to Ld-tRFs. THP-1 cells were cultured in SILAC media and differentiated as described in “Materials and Methods. The “Heavy” cell population was transfected with either Ld-tRF-Asp or Ld-tRF-Leu mimic, while the “Medium” cell population was transfected with either scrambled Ld-tRF-Asp or scrambled Ld-tRF-Leu (control). After 24 h of incubation, cells were processed for MS analysis as described in “Materials and Methods”. Mass spectrometry was performed on a mixture of two lysates, enabling a quantitative comparison between control and treated cells (three independent experiments). The results from the control and treated datasets are summarized in this flowchart. Expression of 20 of the 539 identified proteins differed significantly between the control and Ld-tRF-Asp treatment datasets. Similarly, expression of 18 of the 573 identified proteins differed significantly between the control and Ld-tRF-Leu treatment datasets. (Created with BioRender.com).

We first identified host macrophage proteins that were differentially expressed after delivery of *Leishmania* tRFs. As shown in Fig 1 and S1 Table, we filtered the initial 1749 proteins identified by LC-MS/MS to retain only high-confidence human proteins, resulting in 1736 proteins. For downstream analysis, we retained only proteins identified by two unique peptides and with ratios in two out of three replicates, where variability between ratios was less than 30%. This resulted in 539 proteins in the Ld-tRF-Asp transfected group and 573 proteins in the Ld-tRF-Leu transfected group. We performed one-sample t-tests comparing the normalized (H/M) ratios to one, which represents no change in response to *Leishmania* tRFs. Statistical analysis revealed that 20/539 proteins were significantly modulated (adjusted p-value < 0.1) in the Ld-tRF-Asp transfected group and 18/573 proteins were significantly modulated in the Ld-tRF-Leu group. In the Ld-tRF-Asp transfected group, 18 proteins were upregulated, and 2 were downregulated. In the Ld-tRF-Leu transfected group, 16 proteins were upregulated, and 2 were downregulated. These findings indicate that both Ld-tRFs exert significant differential effects on host protein expression (Table 1).

**Table 1:** List of proteins modulated by *Leishmania* tRFs.

*A: Proteins modulated by Ld-tRF Asp*					
Upregulated					
#	Protein name	Gene name	Number of replicates	Average Fold change	p_value
1	4-trimethylaminobutyraldehyde dehydrogenase	ALDH9A1	2	1.28	0.0709
2	AP-1 complex subunit beta-1	AP1B1	2	1.42	0.0930
3	Asparagine--tRNA ligase, cytoplasmic	NARS	2	1.26	0.0001
4	Cytosol aminopeptidase	LAP3	2	1.30	0.1072
5	Fatty acid-binding protein, adipocyte	FABP4	2	1.34	0.03295
6	Fructose-1,6-bisphosphatase 1	FBP1	2	1.53	0.0015
7	Hematopoietic lineage cell-specific protein	HCLS1	3	1.25	0.0035
8	Hypoxanthine-guanine phosphoribosyltransferase	HPRT1	2	1.30	0.1278
9	Integrin alpha-L	ITGAL	2	1.56	0.0093
10	Leukocyte elastase inhibitor	SERPINB1	2	1.22	0.0361
11	Myeloid cell nuclear differentiation antigen	MNDA	2	1.29	0.0607
12	Nicotinate phosphoribosyltransferase	NAPRT	2	1.38	0.0029
13	Puromycin-sensitive aminopeptidase;Puromycin-sensitive aminopeptidase-like protein	NPEPPS;NPEPPSL1	2	1.59	0.1169
14	Rho GDP-dissociation inhibitor 1	ARHGDIA	2	1.31	0.0656
15	RNA-binding protein 14	RBM14	2	1.22	0.0112
16	Syntaxin-binding protein 3	STXBP3	2	1.29	0.0135
17	Trifunctional purine biosynthetic protein adenosine-3;Phosphoribosylamine--glycine ligase;Phosphoribosylformylglycinamidine cyclo-ligase;Phosphoribosylglycinamide formyltransferase	GART	3	1.57	0.1105
18	UV excision repair protein RAD23 homolog B	RAD23B	2	1.43	0.0265
**Downregulated**					
19	Farnesyl pyrophosphate synthase (FFPS)	FDPS	2	0.78	0.0500
20	Histone H2A.V;Histone H2A.Z;Histone H2A	H2AFV;H2AFZ	2	0.75	0.1029
***B: Proteins modulated by Ld-tRF Leu***					
**Upregulated**					
**#**	**Protein name**	**Gene name**	**Number of replicates**	**Average Fold change**	**p_value**
1	40S ribosomal protein S16 (RPS16)	RPS16	2	1.31	0.0574
2	Calcium-binding protein 39 (CAB 39)	CACYBP	2	1.45	0.1019
3	CD109 antigen	CD109	3	1.33	0.0002
4	Cytochrome c	CYCS	2	1.44	0.0538
5	Cytoplasmic dynein 1 heavy chain 1	DYNC1H1	2	1.30	0.0417
6	Cytosolic non-specific dipeptidase	CNDP2	3	1.36	0.0299
7	DNA topoisomerase 2;DNA topoisomerase 2-beta	TOP2B	2	1.39	0.0137
8	Elongation factor 1-beta	EEF1B2	3	1.47	0.1079
9	Estradiol 17-beta-dehydrogenase 11	HSD17B11	2	1.26	0.1011
10	Fatty acid-binding protein, adipocyte	FABP4	3	1.38	0.0244
11	FUN14 domain-containing protein 2	FUNDC2	2	1.97	0.0807
12	Galectin-3;Galectin	LGALS3	2	1.28	0.0052
13	Heat shock protein beta-1	HSPB1	2	1.30	0.0238
14	Leukocyte elastase inhibitor	SERPINB1	2	1.49	0.1254
***B: Proteins modulated by Ld-tRF Leu***
15	RNA-binding protein FUS	FUS	2	1.25	0.0243
16	V-type proton ATPase subunit B, brain isoform	ATP6V1B2	3	1.33	0.0888
**Downregulated**					
17	Carnitine O-palmitoyltransferase 1, liver isoform	CPT1A	2	0.69	0.0266
18	Long-chain-fatty-acid--CoA ligase 4	ACSL4	2	0.77	0.0267

### Identification of Ld-tRFs modulating macrophages for the potential to create a pro-parasitic environment

**Fig 2:**
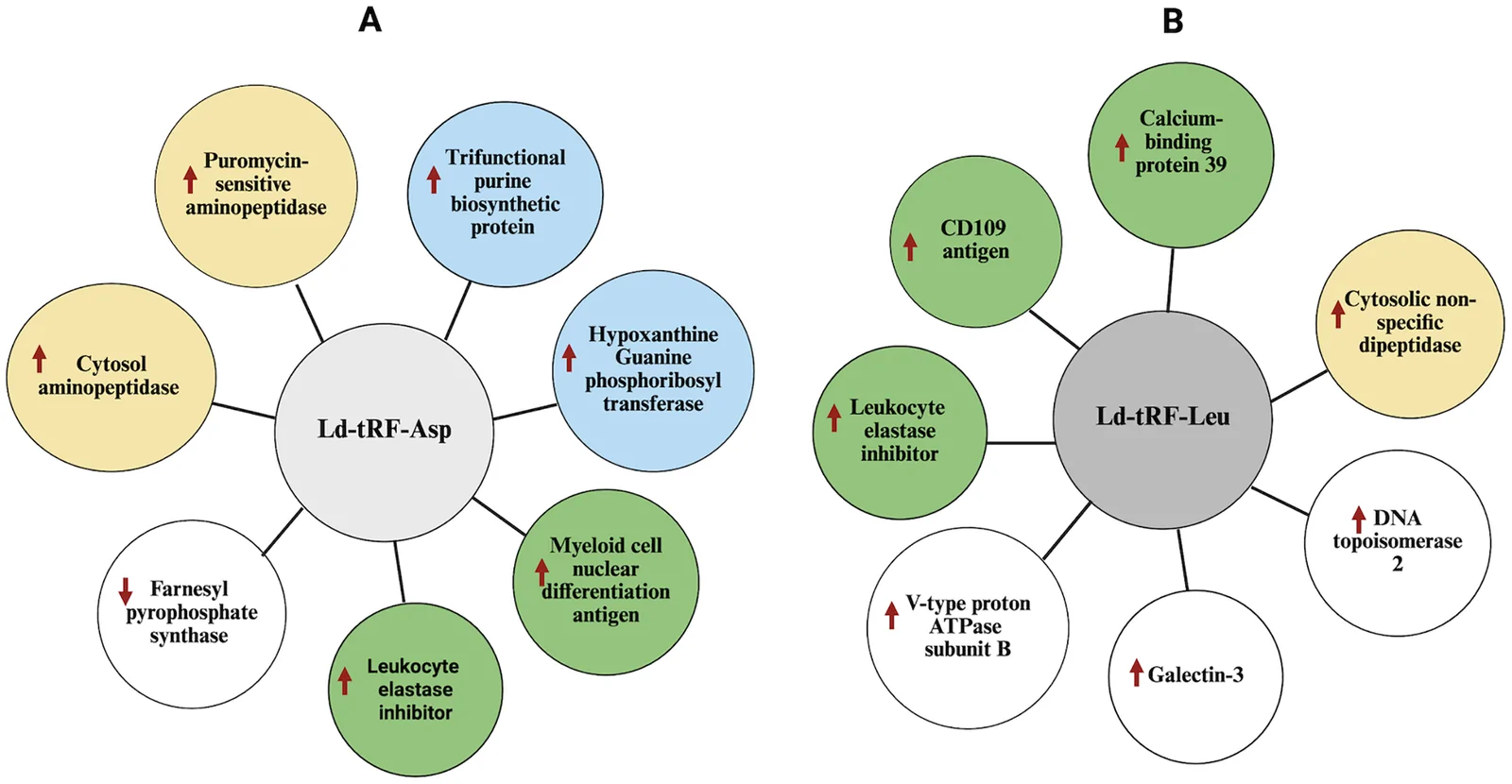
(A) Identification of pro-parasitic macrophage proteins in response to Ld-tRF-Asp exposure. THP-1 cells were prepared for SILAC-based quantitative proteomics. The “Heavy” cell population was transfected with a Ld-tRF-Asp mimic, while the “Medium” cell population was transfected with scrambled Ld-tRF-Asp (control). After 24 h of incubation, cells were processed for MS analysis as described in “Materials and Methods”. Shown are host proteins that promote a pro-parasitic environment. Proteins marked in blue are involved in the purine salvage pathway, green indicates anti-inflammatory polarization, and yellow denotes proteins involved in antigen presentation. (B) Identification of pro-parasitic macrophage proteins in response to Ld-tRF-Leu exposure. In parallel, the “Heavy” cell population was transfected with a Ld-tRF-Leu mimic, while the “Medium” cell population was transfected with scrambled Ld-tRF-Asp (control). After 24 h of incubation, cells were processed for MS analysis as described in “Materials and Methods”. Shown are host proteins that facilitate a pro-parasitic environment. Proteins shown in green are involved in anti-inflammatory polarization, and yellow in antigen presentation. (Created with BioRender.com).

It was of interest to determine how many of the Ld-tRF-modulated proteins are relevant to *Leishmania* pathogenesis. To this end, we searched the literature on the functions of modulated proteins relevant to infection and immunity (Table 2), with particular attention to pro-*Leishmania* effects. For this search, we primarily used PubMed, guided by previous reports of pro-parasitic behaviour in *Leishmania* infection-related studies. 7 of the 20 Ld-tRF-Asp-modulated proteins, 7 of the 18 Ld-tRF-Leu-modulated proteins showed potential to promote *Leishmania* survival. Of these, 6 proteins were upregulated by *Leishmania*-tRFs-Asp (LAP3, HPRT1, SERPINB1, MNDA, NPEPPS, GART) and one was downregulated (FDPS) (Table 2). All 7 proteins were upregulated by *Leishmania*-tRFs-Leu (CACYBP, CD109, CNDP2, SERPINB1, TOP2B, LGALS3, ATP6V1B2) (Table 2). For ease of review, we have also presented the proteins with potential to be pro-*Leishmania* as a bubble diagram (Fig 2).

**Table 2:** List of macrophage proteins modulated by *Leishmania* tRFs with functions relevant to Infection and Immunity.

***A: List of macrophage proteins upregulated by Ld-tRF-Asp***
#	Protein names	Gene name	Known Role in relation to infection and immunity
1	4 trimethylaminobutyraldehyde dehydrogenase	ALDH9A1	ALDH9A1 participates in carnitine biosynthesis by converting 4-trimethylammoniobutanal to 4-trimethylammoniobutanoate [32]. L-carnitine supplementation significantly enhanced immune function in aged rats by improving neutrophil activity and IgA and IgG levels [33].
2	AP-1 complex subunit beta-1	AP1B1	Host AP complexes, including AP-1, have been shown to enhance infection efficiency, as viruses can exploit these complexes through their trafficking functions [34].
3	Cytosol aminopeptidase	LAP3	The upregulation of this dipeptidase in infected cells could limit the availability of peptides for MHC class I presentation, hindering pathogen-specific immune response [35].
4	Fatty acid-binding protein, adipocyte	FABP4	FABP4 is highly expressed in adipocytes and macrophages, and can be secreted into circulation, influencing systemic metabolic and inflammatory processes [36].
5	Fructose-1,6-bisphosphatase 1	FBP1	Fructose-1,6-diphosphate inhibits viral replication by promoting the lysosomal degradation of HMGB1 (High Mobility Group Box 1) and blocking the binding of HMGB1 to the viral genome [37].
6	Hypoxanthine-guanine phosphoribosyltransferase	HPRT1	HGPRT plays a dual role in infection and immunity. It is involved in the purine salvage pathway. In parasitic protozoa, which cannot synthesize purines de novo, upregulation of HGPRT supports parasite survival and replication by synthesizing purines [38]. In the host, high HGPRT expression contributes to immunosuppression [39].
7	Leukocyte elastase inhibitor	SERPINB1	Secretory leukocyte protease inhibitor (SLPI) suppresses the production of pro-inflammatory cytokines like IL-6, TNF-α, and IL-8, thereby dampening the overall inflammatory response. It plays anti-inflammatory and immunomodulatory roles by attenuating NF-kB signaling, a key regulator of pro-inflammatory gene expression [40].
8	Myeloid cell nuclear differentiation antigen	MNDA	MNDA regulates type I interferon production in monocytes via *IRF7,* enhancing antiviral responses [41], and controls neutrophil apoptosis to balance inflammation [42]. In hepatocellular carcinoma, MNDA is highly expressed in M2 macrophages, promoting their polarization and secretion of pro-metastatic factors, contributing to tumor progression [43].
9	Puromycin-sensitive aminopeptidase; Puromycin-sensitive aminopeptidase-like protein	NPEPPS;NPEPPSL1	This protein is involved in maintaining cellular health, is fundamental for a robust immune response. Recent research [44] identified NPEPPS as a key gene upregulated in CD8 + T and NK cells associated with type 2 diabetes and coronary artery disease, highlighting its potential role in immune cell function.
10	Rho GDP-dissociation inhibitor 1	ARHGDIA	RhoGDI1 regulates the Rho family GTPases in immune cells, affecting cell migration, adhesion, and the actin cytoskeleton. Research highlighting the role of RhoGDI1 in the virulence of Cryptococcus neoformans, suggests that these regulators have a role in microbial survival during infection [45].
11	Syntaxin-binding protein 3	STXBP3	Syntaxin-binding protein 3 (STXBP3) regulates cellular secretion pathways important for immunity and inflammation. Mutations in the STXBP3 gene can cause immunodeficiency and inflammatory diseases, highlighting its essential role in both innate and adaptive immune responses. It aids immune cells in releasing signalling molecules, such as cytokines [46].
12	Trifunctional purine biosynthetic protein adenosine-3;Phosphoribosylamine--glycine ligase;Phosphoribosylformylglycinamidine cyclo-ligase; Phosphoribosylglycinamide formyltransferase	GART	Immune cells require purine biosynthesis to proliferate and function, and GART plays a role in these processes [47].
13	UV excision repair protein RAD23 homolog B	RAD23B	RAD23B plays a role in infection and immunity mainly through its involvement in the ubiquitin-proteasome system [48] and by regulating the virulence of pathogens [49].
***B: List of macrophage proteins downregulated by Ld-tRF-Asp***			
**#**	**Protein names**	**Gene name**	**Known Role in relation to infection and immunity**
1	Farnesyl pyrophosphate synthase (FFPS)	FDPS	FPPS enzyme catalyzes the synthesis of farnesyl pyrophosphate (FPP) from its precursors, isopentenyl pyrophosphate (IPP) and dimethylallyl pyrophosphate (DMAPP) in both humans and *Leishmania* [50]. Additionally, FPPS can also play a role in viral inhibition and fungal virulence [51,52].
2	Histone H2A.V;Histone H2A.Z;Histone H2A	H2AFV;H2AFZ	Histone H2A variants, such as H2A.Z, help regulate genes involved in both antiviral defenses and inflammation. They may also act directly against microbes as antimicrobial peptides [53].
***C: List of macrophage proteins upregulated by Ld-tRF-Leu***			
**#**	**Protein names**	**Gene name**	**Known Role in relation to infection and immunity**
1	40S ribosomal protein S16 (RPS16)	RPS16	RPS16 has been shown to regulate inflammation and interact with pathogens [54–56]. The deliberate knockdown of RPS16 expression resulted in increased expression of type I interferon, which subsequently reduced viral replication.
2	Calcium-binding protein 39 (CAB 39)	CACYBP	Enhanced expression of CAB 39 has been linked to the shift from a pro-inflammatory M1 phenotype to an anti-inflammatory M2 phenotype [57].
3	CD109 antigen	CD109	It is known that the tumour-derived CD109 antigen is closely linked to an immunosuppressive response through the reprogramming of macrophages [58].
4	Cytochrome c	CYCS	Cytochrome c has a dual role in infection and immunity: it regulates apoptosis and acts as an immune mediator. Its presence influences both the survival of microbes and the host immune response, as demonstrated in cases of fungal infections [59].
5	Cytoplasmic dynein 1 heavy chain 1	DYNC1H1	DYNC1H1 regulates host immune responses by modulating signalling pathways, including the IFN-γ-JAK-STAT pathway [60].
6	Cytosolic non-specific dipeptidase	CNDP2	CNDP2 plays a role in the breakdown of oligopeptides generated during protein degradation in both healthy and infected cells. As a dipeptidase, increased expression of cytosolic non-specific dipeptidase in infected cells could further degrade oligopeptides, reducing the availability of peptides for MHC class I presentation [35] and impairing the pathogen-specific immune response.
7	DNA topoisomerase 2; DNA topoisomerase 2-beta	TOP2B	Macrophage DNA topoisomerase 2 is essential for DNA replication and segregation [61].
8	Elongation factor 1-beta	EEF1B2	In addition to its established role in protein synthesis, EF-1 beta has been shown to block apoptosis by activating signalling pathways (such as Akt) that promote cell survival, linking it to cancer [62].
9	Estradiol 17-beta-dehydrogenase 11	HSD17B11	HSD17B11 is mainly involved in androgen metabolism, but the broader group of 17β-HSDs, and notably 17β-Estradiol (E2), influences the innate immune response. This illustrates complex interactions between sex hormones and the immune system [63].
10	Fatty acid-binding protein, adipocyte	FABP4	AFABP is an important contributor to inflammation, particularly among macrophages and adipocytes [64].
11	FUN14 domain-containing protein 2	FUNDC2	As a mitochondrial protein, FUNDC2 is involved in mitophagy, a crucial process for maintaining cellular health by removing damaged mitochondria. Proper mitophagy is essential for controlling inflammatory responses during infection [65].
12	Galectin-3; Galectin	LGALS3	Galectin-3 has been implicated in anti-apoptotic activity [66] and the suppression of autophagy [67].
13	Heat shock protein beta-1	HSPB1	Heat Shock Protein beta-1 (HSP27) is a small heat shock protein (sHSP) that functions as a molecular chaperone. Depending on the situation, it can both promote or inhibit infection and immunity. HSP27 is also involved in the inflammatory and immune responses to pathogens [68].
14	Leukocyte elastase inhibitor	SERPINB1	Secretory leukocyte protease inhibitor (SLPI) suppresses the production of pro-inflammatory cytokines like IL-6, TNF-α, and IL-8, thereby dampening the overall inflammatory response. It plays anti-inflammatory and immunomodulatory roles by attenuating NF-kB signaling, a key regulator of pro-inflammatory gene expression [40].
15	RNA-binding protein FUS	FUS	FUS has been shown to promote the expression of type I interferons and pro-inflammatory cytokines in response to viral or bacterial infections [69].
16	V-type proton ATPase subunit B, brain isoform	ATP6V1B2	The brain-specific V-type proton ATPase (V-ATPase) subunit B is essential for maintaining lysosomal acidification [70]. *Leishmania* resides within the acidic environment of phagolysosomes in infected cells. Upregulation of this protein in leishmania-infected macrophages may help preserve the acidic environment of phagolysosomes.
***D: List of macrophage proteins downregulated by Ld-tRF-Leu***			
**#**	**Protein names**	**Gene name**	**Known Role in relation to infection and immunity**
1	Carnitine O-palmitoyltransferase 1, liver isoform	CPT1A	Carnitine O-palmitoyltransferase 1 is essential for infection and immunity because it regulates energy metabolism in immune cells, especially neutrophils [71].
2	Long-chain-fatty-acid--CoA ligase 4	ACSL4	Long-chain acyl-CoA synthetase 4 (ACSL4) plays a complex role in infection and immunity, primarily by regulating lipid metabolism and phospholipid remodelling in immune cells, such as macrophages and neutrophils [72].

### Ago protein-containing complexes isolated from *Leishmania*-infected cells revealed the selective presence of tRF-Asp and tRF-Leu

**Fig 3:**
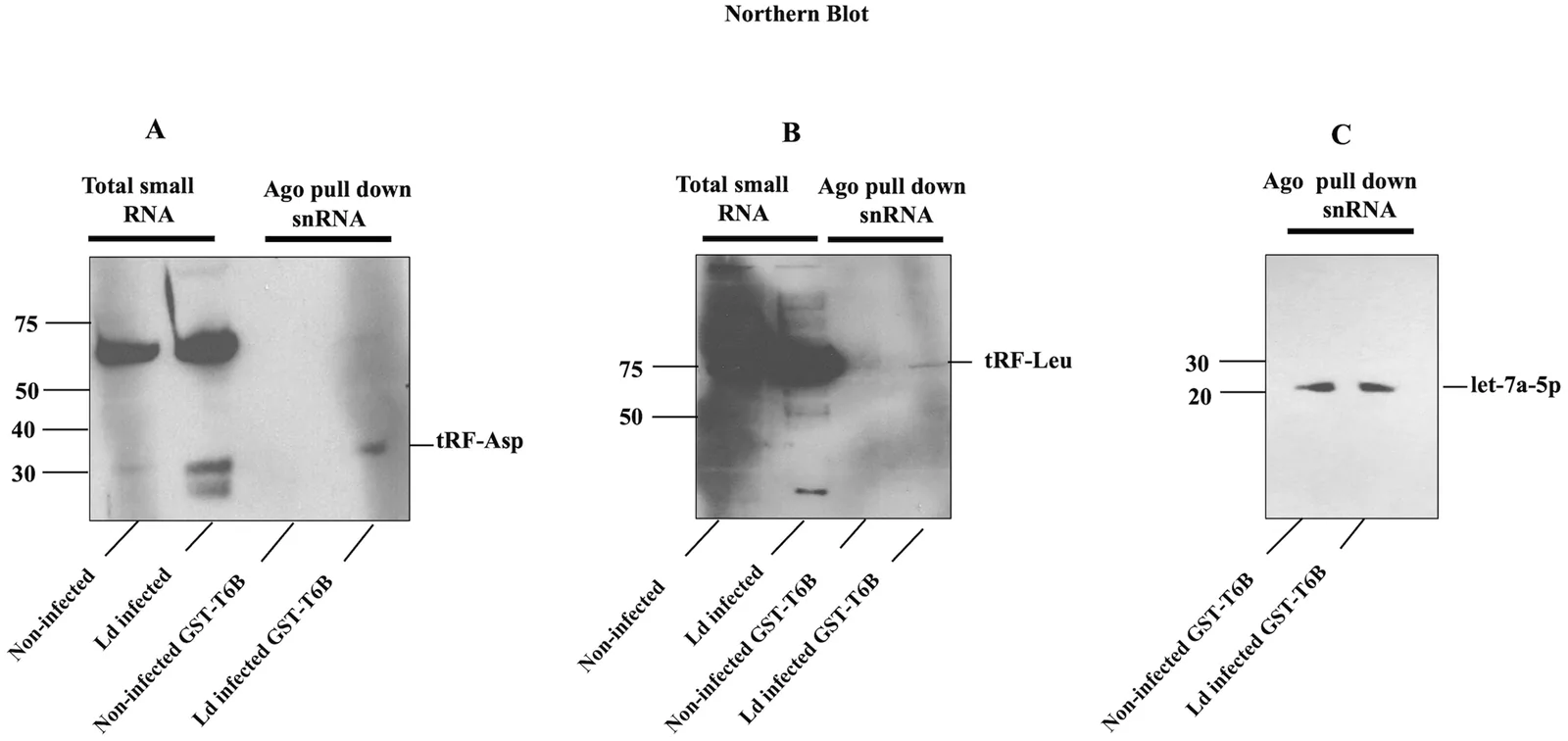
GST-T6B affinity isolation of Ago complexes and associated RNAs from control and *L. donovani*-infected dTHP-1 cells. Equal amounts of recombinant GST (control) or GST-T6B proteins were loaded onto glutathione-agarose beads as described in ‘Materials and Methods”. dTHP-1 cells, infected or not with *L. donovani*, were processed 24 h post-infection for whole cell lysates and separately incubated with GST-T6B affinity. Beads were washed and used to extract bound RNAs, which were then separated on a 15% denaturing polyacrylamide TBE/7M urea gel. RNAs were transferred onto a positively charged Hybond N+ nylon membrane and analysed for the presence of tRFs by Northern blotting as described in “Materials and Methods”. Northern blots were probed for the presence of either tRF-Asp (panel A) or tRF-Leu (panel B) using specific labeled probes. The presence of an abundant endogenous miRNA, let-7a-5p, was used as a positive control to assess the specificity of GST-T6B (panel C). The results shown represent one of three independent experiments.

As shown above, *Leishmania* tRFs have the potential to create a pro-parasitic environment that supports *Leishmania* growth and persistence. This exciting finding encouraged us to investigate the molecular mechanisms by which *Leishmania* tRFs modulate host proteins. Regarding the functions of tRNA fragments, it is now clear that tRFs can act through multiple mechanisms, without following a single consistent pattern [73,74]. These functions may be Ago-dependent [75] or Ago-independent [73]. As mentioned earlier, previous studies from this lab showed that *L. donovani* infection led to global downregulation of host miRNAs [76] and enhanced the abundance of host macrophage Ago1 protein and its incorporation into RISC complexes [19]. We therefore hypothesized that *Leishmania* tRFs mediate their action by loading onto host Ago proteins, preferentially using Ago1. To test whether *Leishmania*-derived tRNA fragments—Ld-tRF-Asp and Ld-tRF-Leu—are loaded onto host Ago proteins, we used the Ago-APP affinity purification previously validated in our laboratory [18]. This method isolates active RISC complexes containing Ago proteins along with their associated small non-coding RNAs. Cytoplasmic fractions from uninfected and *L. donovani*–infected dTHP-1 cells were used to selectively capture Ago-containing complexes, followed by processing of beads for small RNA isolation as described in the “Materials and Methods”. Northern blot analysis of the eluates revealed that both tRF-Asp and tRF-Leu are selectively enriched on Ago proteins in infected cells (Fig 3, panels A & B). The expected size range for mature tRFs is typically 14–40 nt [77]; however, the tRF-Leu species detected migrated at approximately 75 nt. This likely reflects hybridization of the probe to full length tRNA or larger tRNA-derived fragments. In contrast, the tRF-Asp species migrated at ~32 nt, which falls within the expected size of a canonical tRF. Let-7a-5p was used as a positive control for Ago loading and showed robust enrichment in the Ago-APP eluates (Fig 3, panel C).

### *Leishmania* tRF showed a preference for interaction with Ago 1

**Fig 4:**
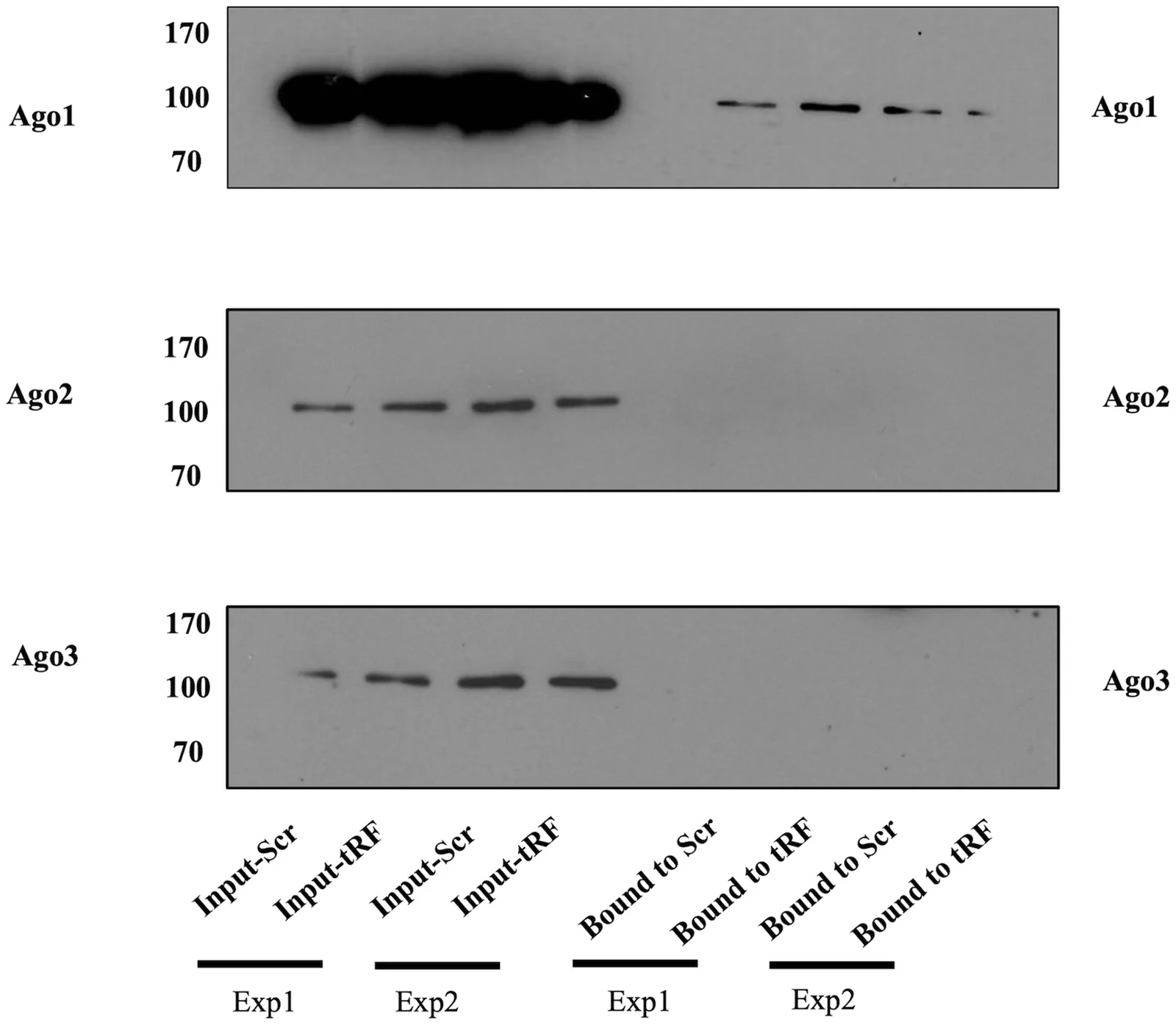
Isolation of Ld-tTRF-interacting proteins *in vivo.* A synthetic biotinylated L-tRF-Leu (50 nM) and a corresponding biotinylated scrambled tRF-Leu (50 nM) were separately transfected into dTHP-1 cells. After 24 h of oligo treatment, the RNA pull-down assay was performed as described in “Materials and Methods”. Bound Ago complexes were captured on streptavidin-conjugated magnetic beads. After washing to remove non-specific interactions, the beads were analyzed by Western blotting with the indicated Ago-specific antibodies to detect Ago proteins. The results shown represent two independent experiments.

Because the Ago-APP method captures all Ago proteins without isoform specificity, we next sought to determine whether *Leishmania* tRFs preferentially load onto a particular host Ago protein. For this investigation, a synthetic biotinylated L-tRF-Leu mimic was transfected into dTHP-1 cells for 24 h (S2A Table in S2 File). A corresponding biotinylated scrambled tRF was used as a control (Table S2A in S2 File). Whole cell lysates were prepared from THP-1 cells as described in “Materials and Methods”, precleared with streptavidin beads alone to remove potential non-specific binding proteins, and then the cleared supernatant was incubated with streptavidin magnetic beads to pull down the biotinylated tRF-Leu along with interacting proteins. The associated proteins were subsequently analyzed by Western blotting using Ago-specific antibodies. As shown in Fig 4, tRF-Leu demonstrated a strong and consistent interaction with host Ago1 in whole-cell lysates. Ago2 and Ago3 binding could not be detected. Because Ago1 does not require perfect sequence complementarity for small RNA binding, a scrambled tRF oligo was used as a positive control for expected Ago1 binding.

Given the observed potential Ago1 association, we next assessed whether tRF-Leu and tRF-Asp share complementarity with gene sequences of proteins differentially expressed upon transfection of L-tRF-Leu and L-tRF-Asp in THP-1 cells (Tables 1 and 2). To assess potential direct binding, we ran RNAhybrid v2.1.2 [78]; within an Anaconda/Python environment. Hybridization analyses were conducted across 5′UTR, coding sequence (CDS), and 3′UTR regions of target transcripts. Predicted tRF–mRNA interactions were filtered using a minimum free energy (MFE) threshold of <−20 kcal/mol and a significance cut-off of p < 0.05. Using these criteria, multiple high-confidence interactions were identified for both tRFs across several differentially expressed genes (S2B Table in S2 File). Interestingly, RNAhybrid predicted energetically favourable hybridization between tRF-Leu binding to the 5′UTR of *CPT1A* (MFE = −33.7 kcal/mol), *SERPINB1* (MFE = −26.1 kcal/mol), *LGALS3* (MFE = −23.3 kcal/mol), *TOP2B* (MFS = −33.5 kcal/mol), *ATP6V1B2* (MFE = −19.8 kcal/mol) and *FUNDC2* (MFE = −23.5 kcal/mol) and tRF-Asp binding to the 5′UTR of *AP1B1* (MFE = −35.6 kcal/mol), *ARHGDIA* (MFE = −30.6 kcal/mol) and *STXBP3* (MFE = −31.8 kcal/mol) and the CDS of *H2AZ2* (MFE = −36.2 kcal/mol) (S2B Table in S2 File and S2 Fig. in S3 File). Previous studies have shown that small non-coding RNA interactions within the 5′UTR can modulate translation either positively or negatively [79,80], distinct from the canonical 3′UTR-associated silencing mechanism.

Together, the quantitative proteomics analysis of *Leishmania* tRFs-modulated proteins reveals that a significant number of Ld tRFs-modulated proteins have potential implications in *Leishmania* pathogenesis, based on previously published studies. How many of the remaining *Leishmania*-tRFs-modulated host proteins play a role in *Leishmania* infection-related activities remains to be investigated. RNA pulldown assays clearly indicate that tRFs preferentially bind to Ago1. Whether Ld-tRFs perform functions beyond engaging Ago1 needs to be elucidated.

## Discussion

It is increasingly clear that tRFs play diverse roles in normal and diseased conditions, as recently reviewed [81–83]. One notable feature of tRFs is their packaging into exosomes. Emerging evidence indicates that tRFs in extracellular vesicles (EVs) function as regulatory molecules in various cellular activities and are crucial to cell-to-cell communication [84]. Recently, it has become evident that pathogen exosomal tRNA fragments play significant regulatory roles in the complex interactions between hosts and pathogens [85], serving as key mediators of cross-kingdom communication and manipulating host cell functions [86]. It has been suggested that tRFs delivered by microbial EVs to host cells represent a widespread mechanism for hijacking host cell biology to promote microbial survival [86]. In light of this emerging hypothesis, this study was initiated to explore the effects of *Leishmania* exosomal tRFs on host cell biology by examining potential changes in the host proteasome in THP-1, a model host cell line [87,88]. For this study, we selected Ld-tRF-Asp and Ld-tRF-Leu because they are the most abundant in both *L. donovani* and *L. brazilensis* exosomes in our previous work [12].

SILAC-based quantitative proteomics was used to investigate the roles of *Leishmania* exosomal tRFs in host macrophages. The core advantage of SILAC over other quantitative proteomic methods (e.g., iTRAQ, TMT, ICAT) and label-free quantification is that samples can be mixed at the lysate level, minimizing quantitative errors that arise when handling multiple samples in parallel. In addition, cell labelling eliminates errors caused by serum contamination in the media. For this analysis, synthetic Ld-tRF-Asp and Ld-tRF-Leu were delivered separately to macrophages already labelled with SILAC isotopes for 24 h, followed by proteomic analysis of whole-cell lysates, which revealed differentially expressed proteins in response to Ld-tRFs. Ld-tRF-Asp significantly modulated 20 host proteins, whereas Ld-tRF-Leu significantly affected 18 host proteins. Interestingly, 7 of the Ld-tRF-Asp-modulated proteins and 7 of the Ld-tRF-Leu-modulated proteins showed potential pro-*Leishmania* effects (Fig 2). Their functions have previously been implicated in *Leishmania* infection-related processes*.*

For example, Ld-tRF-Asp-mediated upregulation of **cytosolic aminopeptidase** [35] and **puromycin-sensitive aminopeptidase** [89] in macrophages could limit the availability of peptides for MHC class I presentation, potentially hindering pathogen-specific immune responses. Therefore, upregulation of these proteins, leading to reduced peptide loading on MHC class I in *Leishmania-*infected cells, could be one mechanism by which *Leishmania* evades the host immune response. **The leukocyte elastase inhibitor,** which possesses anti-inflammatory and immunomodulatory properties, is upregulated in both Ld-tRF-Asp-treated and Ld-tRF-Leu-treated cells. This inhibitor appears to exert these effects by attenuating NF-kB signaling [40]. *Leishmania* parasites actively manipulate the host macrophage’s NF-kB pathway to inhibit pro-inflammatory responses and promote immunosuppressive responses. This manipulation is achieved through various mechanisms, including increasing the expression of NF-κB inhibitors, decreasing the activity of activators, and using parasite-derived enzymes to degrade NF-κB subunits [90]. *Leishmania* are purine auxotrophs and must salvage purines from the host to support their survival and replication [47]. **Hypoxanthine-guanine phosphoribosyltransfe**rase (HGPRT) is essential for salvaging purine bases [38]. **Trifunctional purine biosynthetic protein** GART is another upregulated protein in Ld-tRF-Asp-treated cells, which plays a role in purine synthesis [47]. It is known that *Leishmania* promotes alternative activation (anti-inflammatory polarization) in infected macrophages [91] to create an immunosuppressive environment. MNDA is highly expressed in alternatively activated macrophages, promoting their polarization [43]. Therefore, upregulation of **Myeloid cell nuclear differentiation antigen** in Ld-tRF-Asp-treated cells could also have a pro-parasitic effect. **Farnesyl pyrophosphate synthase (FFPS)** is downregulated in Ld-tRF-Asp-treated THP-1 cells. *Leishmania* rely on the FPP produced by FPPS for essential metabolic functions [50]. It seems reasonable to assume that *Leishmania* downregulates host FPPS to obtain more FPP precursors for its own FPPS.

Interestingly, 6 of the Ld-tRF-Leu-upregulated proteins also showed potential for pro-*Leishmania* effects. For example, enhanced expression of **calcium-binding protein 39** has been linked to the promotion of an anti-inflammatory phenotype [57]. As discussed above, *Leishmania* promotes anti-inflammatory polarization that favours its persistence [92]. Interestingly, Ld-tRF-Leu upregulates tumour-derived **CD109 antige**n, which is closely linked to an immunosuppressive response by reprogramming macrophages and shifting the immune balance towards an anti-inflammatory state [58]. In this context, it is known that *Leishmania* promotes an immunosuppressive environment to establish chronic infection [93]. Therefore, it is reasonable to assume that *Leishmania* exploits the immunosuppressive state resulting from CD109 overexpression to promote its survival. The increased levels of **cytosolic non-specific dipeptidase** caused by Ld-tRF-Leu could lead to a reduced number of peptides available for MHC class I presentation [35]. This decline in antigen presentation might weaken pathogen-specific immune responses, favouring *Leishmania* infection.

Upregulation of host **DNA topoisomerase 2-beta** by Ld-tRF-Leu treatment may also support the survival and growth of *Leishmania* amastigotes. Macrophage DNA topoisomerase 2 is essential for DNA replication and segregation [61]. This could provide a safe and stable environment for *Leishmania* amastigote propagation. **Galectin-3** has been implicated in anti-apoptotic activity [66] and in the suppression of autophagy [67]. The upregulation of Galectin-3 may be relevant to pathogenesis, as both anti-apoptotic and autophagy-inhibiting activities are influenced by *Leishmania* infection [94,95]. Upregulated host brain-specific **V-type proton ATPase (V-ATPase) subunit B** is crucial for maintaining lysosomal acidification [70], and *Leishmania* resides within the acidic environment of phagolysosomes in infected cells. Thus, upregulation of V-ATPase may help preserve the acidic environment of *Leishmania-*containing phagolysosomes.

Taken together, it appears that delivering Ld-tRFs to macrophages modulates multiple host proteins. These modulated protein functions can be categorized into three broad activities: enhancing the salvage pathway of purines, promoting anti-inflammatory polarization, and reducing peptide loading onto MHC class I. All three activities have the potential to create an environment favourable to the survival and growth of *Leishmania.*

Our study showed that one mechanism by which *Leishmania* tRFs affect host biology could involve Ago proteins. Notably, Ago-bound Ld-tRF-Leu showed a much higher molecular weight than expected on the Northern blot in infected cells (Fig 3), suggesting likely hybridization of the probe with full-length tRNA or larger tRNA fragments. Both Dicer-dependent and independent tRFs have been shown to bind Ago to form RISC, which is guided to target mRNAs at sites of partial complementarity, resulting in translational repression and mRNA decay [96]. The sequence similarity between *L. donovani* and human tRNA-Leu is approximately 85%, whereas the similarity between their tRNA-Asp sequences is approximately 90%. Due to these high similarities, the Northern blot cannot distinguish between parasite-derived and host-derived tRF-Leu or tRF-Asp. However, seemingly selective loading of Ld-tRFs onto Ago within the RISC complex remains to be investigated despite the high degree of similarity with corresponding human tRFs. One possibility is that Ld-tRFs could be differentially post-transcriptionally modified, facilitating their binding to Ago proteins compared to human tRFs.

Our *in vivo* RNA pulldown binding assay (Fig 4) showed preferential binding to Ago1. An earlier study from Kumar et al. revealed a clear preference for 5’-tRFs and 3’-tRFs to associate with Ago1, 3, and 4 rather than Ago2 [97]. Additionally, another study involving mammalian cells infected with Epstein-Barr virus showed that sncRNAs other than miRNAs were specifically loaded onto Ago1, but not Ago2 [98]. More recent studies, however, have shown that tRFs can associate with multiple Argonaute proteins in a context-dependent manner, including Ago2-associated loading and regulatory activity [75,96,99]. Therefore, our data support preferential Ago1 association in our experimental system, rather than exclusive Ago1 loading.

Our results predicted that tRF-Leu and tRF-Asp forms energetically favorable hybrids with gene sequences of proteins differentially expressed upon transfection of L-tRF-Leu and L-tRF-Asp in THP-1 cells (S2B Table in S2 File and S2 Fig. in S3 File) and we found statistically significant binding of tRF-Leu to the 5′UTR of *CPT1A*, *SERPINB1*, *LGALS3*, *TOP2B*, *ATP6V1B2* and *FUNDC2*, and tRF-Asp binding to the 5′UTR of *AP1B1*, *ARHGDIA* and *STXBP3* and the CDS of *H2AZ2* (S2B Table in S2 File and S2 Fig. in S3 File). We acknowledge that although computational methods for predicting biological functions are effective, they have limitations. Therefore, these predictions should be validated through experiments before undertaking detailed studies. Biological functions are highly complex and can behave unpredictably. A reporter assay, where the reporter and tRF mimics are co-transfected, could effectively confirm the predicted interaction between Ld-tRFs and mRNAs [96].

It is now clear that tRFs can perform functions through diverse mechanisms, without following a single consistent pattern [73,74]. In a *Leishmania*-related organism, *Trypanosoma brucei,* the 3’ half of tRNA-Thr stimulates overall protein translation by interacting with ribosomes [100]. tRFs can also silence genes by base pairing with target mRNAs. Shorter tRFs, similar in size to microRNAs, have been observed to bind Ago proteins, as microRNAs do [97]. In fact, tRFs have been identified within RISC alongside their complementary target mRNAs [96,97]. tRFs can also function by interacting with RNA-binding proteins (RBPs) other than Ago [101,102]. Our study does not rule out the possibility that some of the Ld-tRFs-mediated effects observed could be Ago-independent.

We note that most macrophage proteins were upregulated, whereas only a few were downregulated in response to Ld-tRFs. This study does not explain the mechanism (s) underlying this skewed response. However, this response could be due to multiple factors. For example, some tRFs occupy or sequester the available pool of Ago, preventing Ago from binding to other small RNAs, such as microRNAs (miRNAs), which are essential for gene silencing [103]. When Ago is sequestered, the usual degradation of existing mRNAs stops, resulting in a broad buildup—and therefore an increase—of many transcripts. It is also known that many tRFs target upstream regulators rather than binding to mRNA directly. For example, inhibiting a single transcriptional repressor with a tRF can significantly increase the expression of many downstream target genes. In contrast, if a tRF influences just a few crucial activators, only a limited number of genes will be downregulated [104,105]. Additionally, enhanced ribosomal protein synthesis induced by tRF’s could be responsible for the observed upregulation of proteins. A notable study by Kim et al. demonstrated that a 22 nt tRF called 3′ tRF^LeuCAG^ enhances translation by aiding ribosome protein biogenesis [106]*.*

We recognize that limitations in our study could influence the breadth of applicability of our findings and their interpretation. First, using a single cell line (THP-1) and a single *Leishmania* species (*L. donovani*) may limit the relevance of our results to other cell types or tissues. THP-1 cells are a common human monocytic leukemia cell line used to model macrophage infections, but they have limitations in *Leishmania* research compared to primary human macrophages or in vivo models. Since they are not primary cells, their signaling pathways, gene expression, and metabolism may not accurately represent those of normal human macrophages. This can lead to differences in cytokine responses or baseline activation that primary monocyte-derived macrophages (MDMs) wouldn’t exhibit. Additionally, THP-1 cells depend on an artificial differentiation agent [107]. The presence of additional non-coding RNAs in *Leishmania* exosomes could also affect the proteomic landscape of host cell biology. We also note that a more lenient p-value threshold was used to identify broader trends in the data. Although using a stricter p-value reduces false positives, the relaxed threshold increases statistical power and decreases the risk of false negatives in this exploratory phase. Thus, these proteomic results should be confirmed using additional techniques, such as Western blotting and transcriptional measurements (e.g., qRT-PCR).

**Fig 5:**
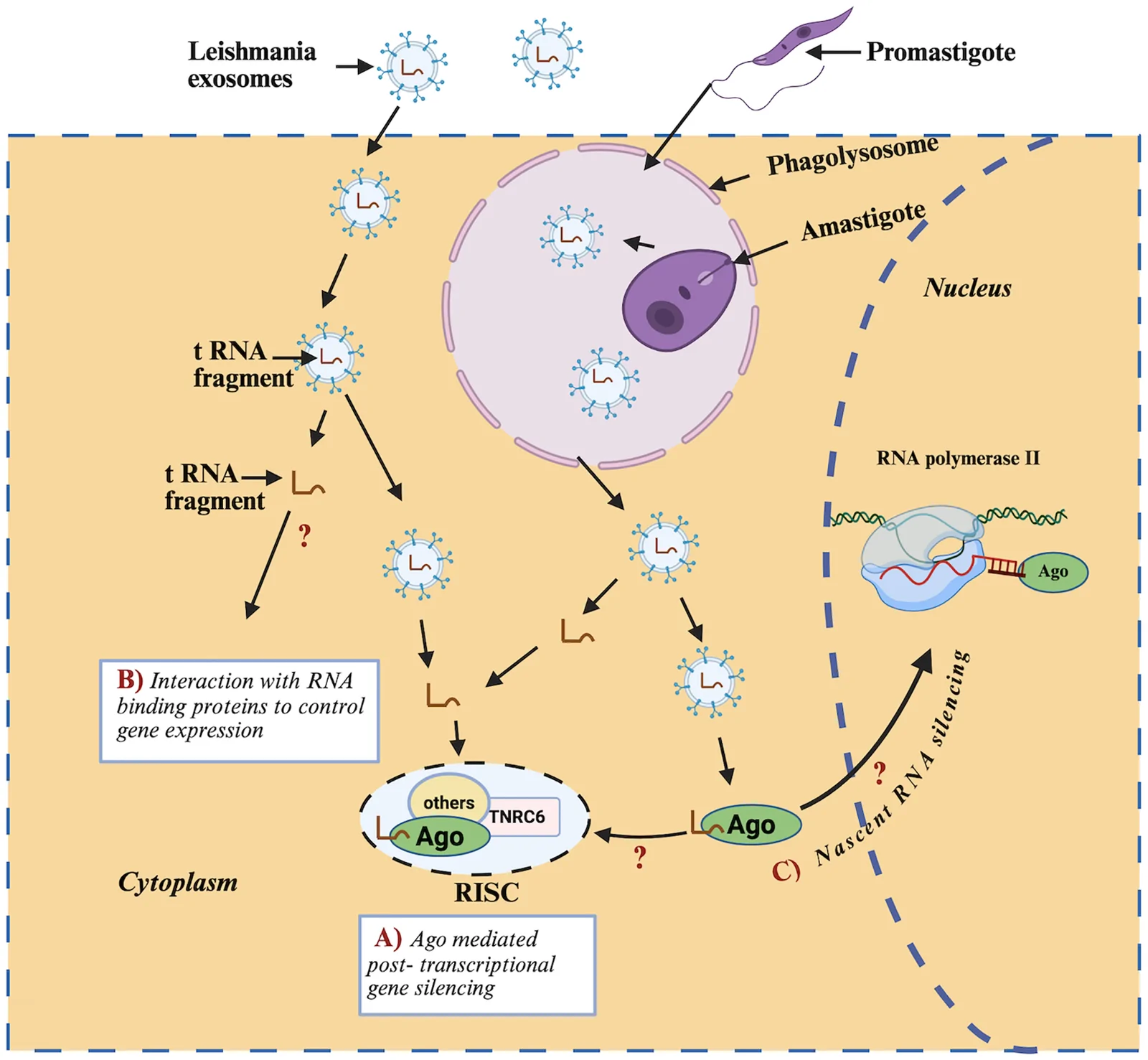
Hypothetical model of *Leishmania* tRFs mediating modulation of host macrophage gene expression. *Leishmania* tRFs are delivered into the host cell cytosol via exosomes secreted within the phagolysosome of the *Leishmania*-infected host cell or via secreted exosomes released by infected bystander cells. *Leishmania* tRFs released from exosomes could be preferentially loaded onto host Ago1, which does not require complete complementarity to target mRNA. Ago1, together with other proteins, forms the RISC. The RISC then regulates gene expression post-transcriptionally by targeting mRNAs. tRF-loaded Ago1 could also enter the nucleus, leading to nascent RNA silencing. As Ago-independent mechanisms, tRFs directly interact with other RNA-binding proteins to regulate host gene expression. (Created with BioRender.com).

In summary, our study demonstrates that delivering specific *Leishmania* tRFs to host THP-1 cells results in significant changes in the host proteome. Interestingly, most of the modulation of host proteins appears to create a pro-*Leishmania* environment that promotes *Leishmania* survival. Additionally, we show that Ld-tRFs tend to bind host Ago1, indicating that Ago-mediated gene regulation is one mechanism involved. The possibility of other mechanisms is shown in Fig 5. Overall, the results presented here contribute to our understanding of the regulatory functions of *Leishmania* tRFs in host biology. A deep quantitative and qualitative understanding of the effects of *Leishmania* tRFs on host cell biology could reveal a previously unknown role for tRFs in disease progression. Understanding how pathogen tRFs influence host gene expression provides new insights into the complex mechanisms of *Leishmania* pathogenesis and could inform future treatments that target these regulatory molecules to manage leishmaniasis.

## Supporting information

S1Fig Schematic diagram of metabolic SILAC quantitative proteomics of Ago-complexes. THP-1 cells were cultured in medium and heavy SILAC media. Labeled THP-1 cells were differentiated with PMA and subsequently transfected with Ld-tRF-Asp and Ld-tRF-Leu mimics or corresponding scrambled oligos for 24 hours as indicated. Post-transfection, whole cell lysates were processed for SILAC-based quantitative proteomics as described in “Materials and Methods”.
(TIF)

S1 Table dTHP-1 proteins modulated by *Leishmania* tRFs. (XLSX)

S1 File Raw Images. (PDF)

S2 File Tables S2A & S2B. S2A.Sequence of oligos used for biotin-RNA pull down assays. S2B. Significant predicted interactions between the *Leishmania* tRFs and target genes identified using RNAhybrid.
(DOCX)

S3 File S2 Fig. Predicted interactions between the *Leishmania* tRFs and their target genes identified using RNAhybrid with target binding coordinates indicated. (PDF)
