# Supplementary material for: Leishmania exosomal tRNA-derived small non-coding RNA fragments modulate host THP-1 derived macrophage proteins: A quantitative proteomic analysis
Source: PLoS One. 2026 Aug 19;21(8):e0356144. doi: 10.1371/journal.pone.0356144 (PMC13489484; doi:10.1371/journal.pone.0356144)
Supplement: Supplementary file 1 [file pone.0356144.s003.pdf]

Fig 3A & B: Northern blot analysis of the small RNA isolated from dTHP-1 cells.

15% denaturing polyacrylamide TBE/7M urea gel
Hybond N+ nylon membrane

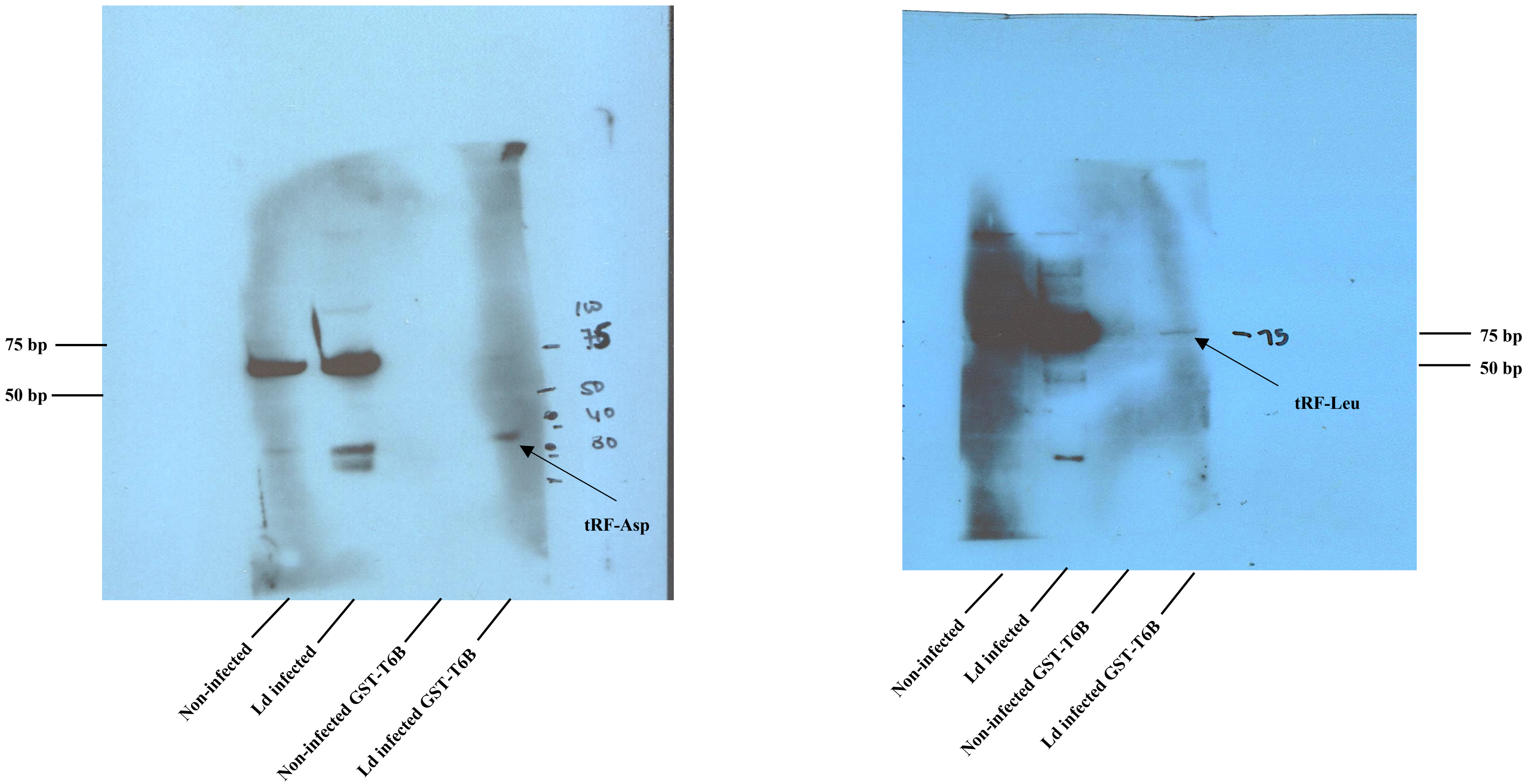

Image captured via ECL and X-ray film

Fig 3C: Northern blot analysis of the small RNA isolated from dTHP-1 cells.

15% denaturing polyacrylamide TBE/7M urea gel

Hybond N+ nylon membrane

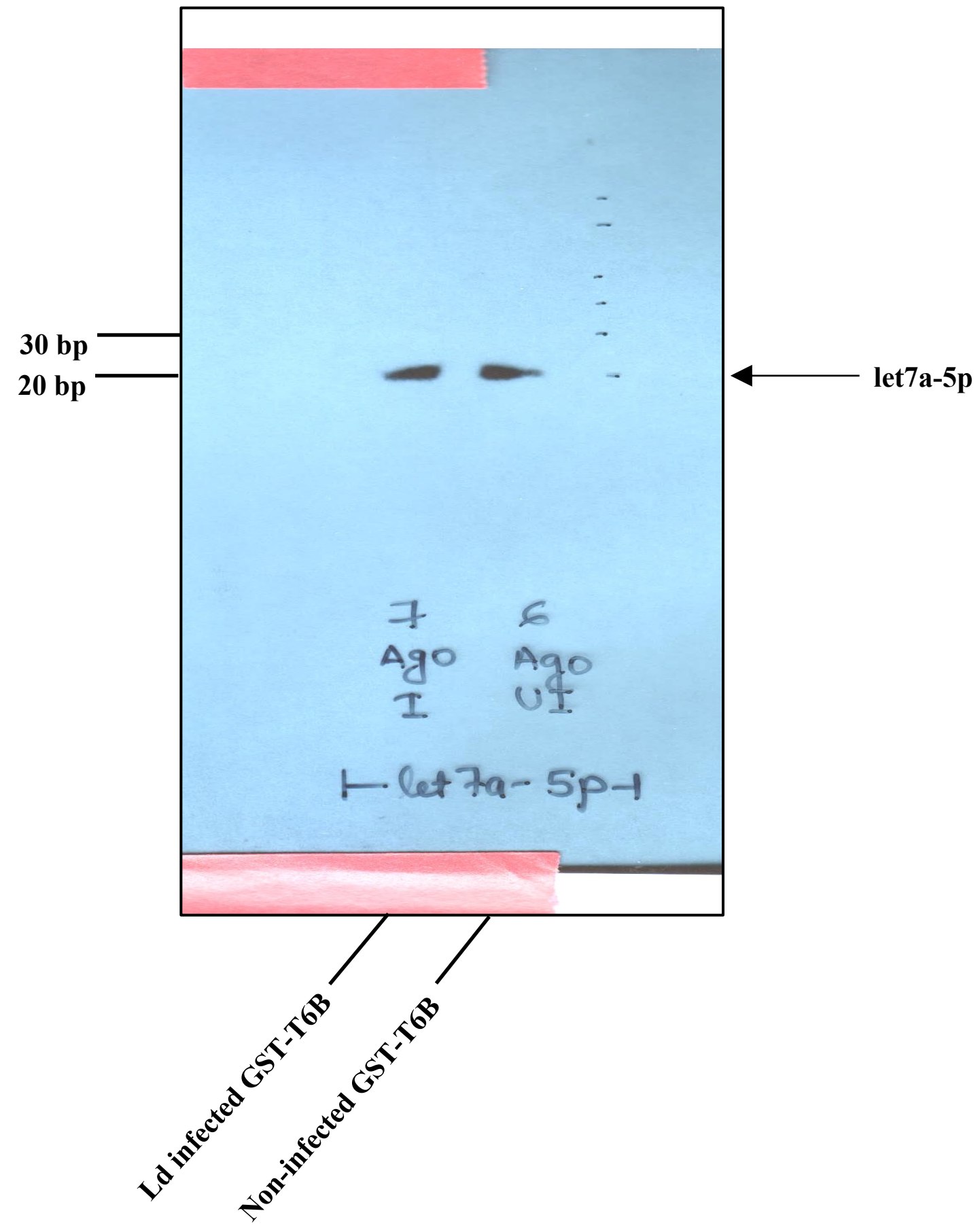

Image captured via ECL and X-ray film

Fig 4A Western blot to check binding of Argonaute proteins to biotinylated Leishmania tRF-Leu mimic
8% Tris-Glycine SDS-PAGE
Nitrocellulose

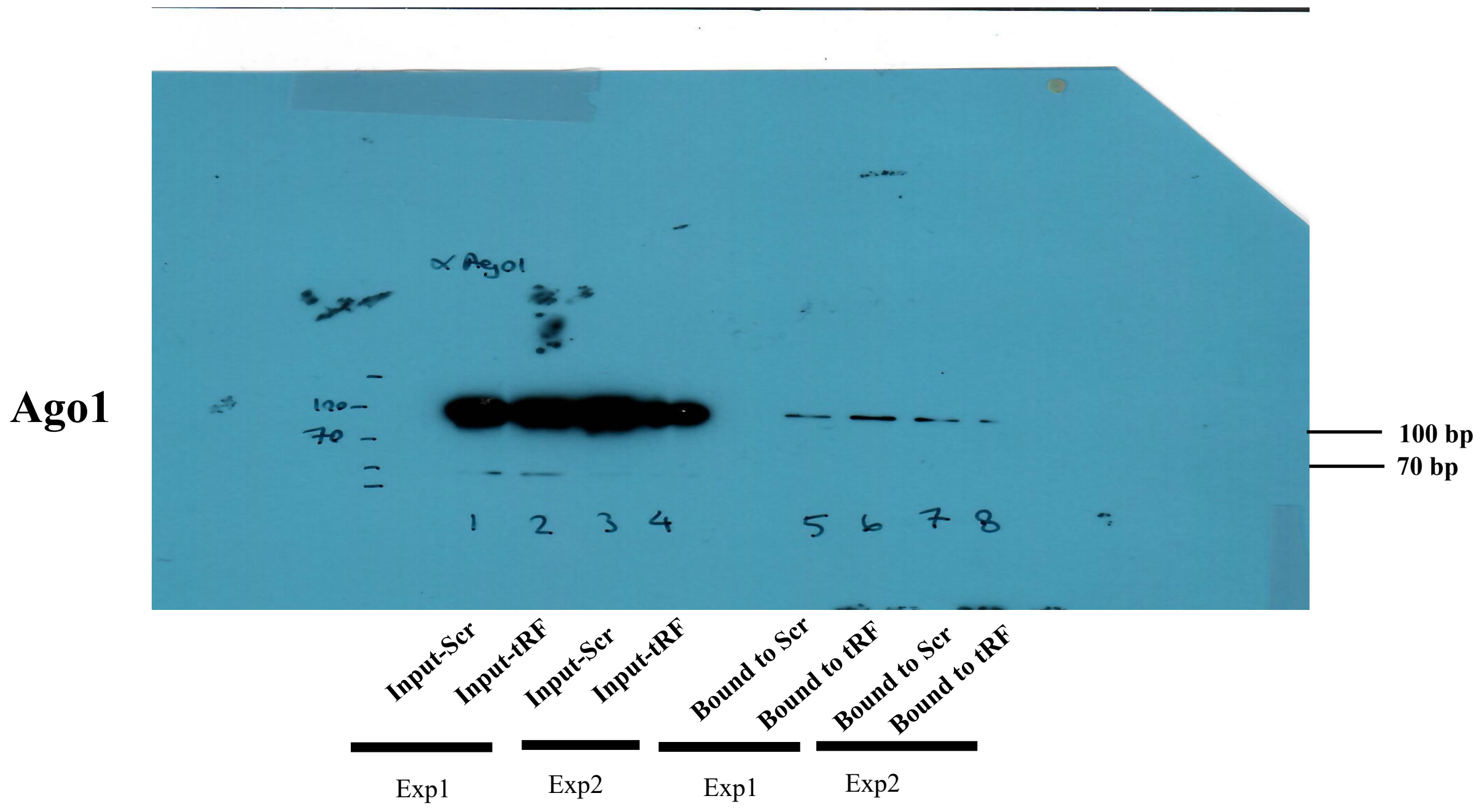

Image captured via ECL and X-ray film

Fig 4B Western blot to check binding of Argonaute proteins to biotinylated Leishmania tRF-Leu mimic
8% Tris-Glycine SDS-PAGE
Nitrocellulose

Ago2

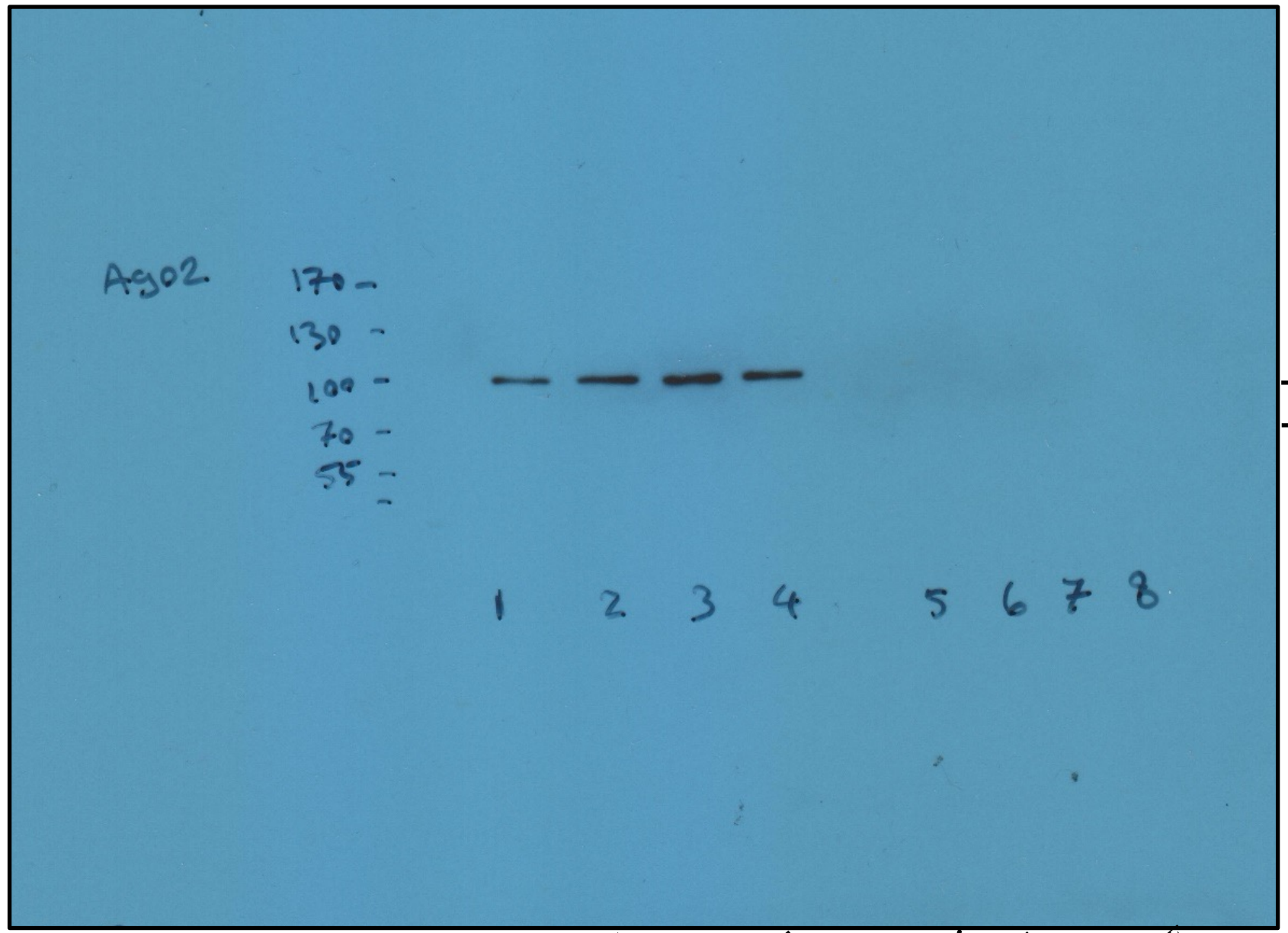

100 bp
70 bp

Input-Scr | Input-tRF | Input-Scr | Input-tRF | Bound to Scr | Bound to tRF | Bound to Scr | Bound to tRF

Exp1 | Exp2 | Exp1 | Exp2

Image captured via ECL and X-ray film

Fig 4C Western blot to check binding of Argonaute proteins to biotinylated Leishmania tRF-Leu mimic
8% Tris-Glycine SDS-PAGE
Nitrocellulose

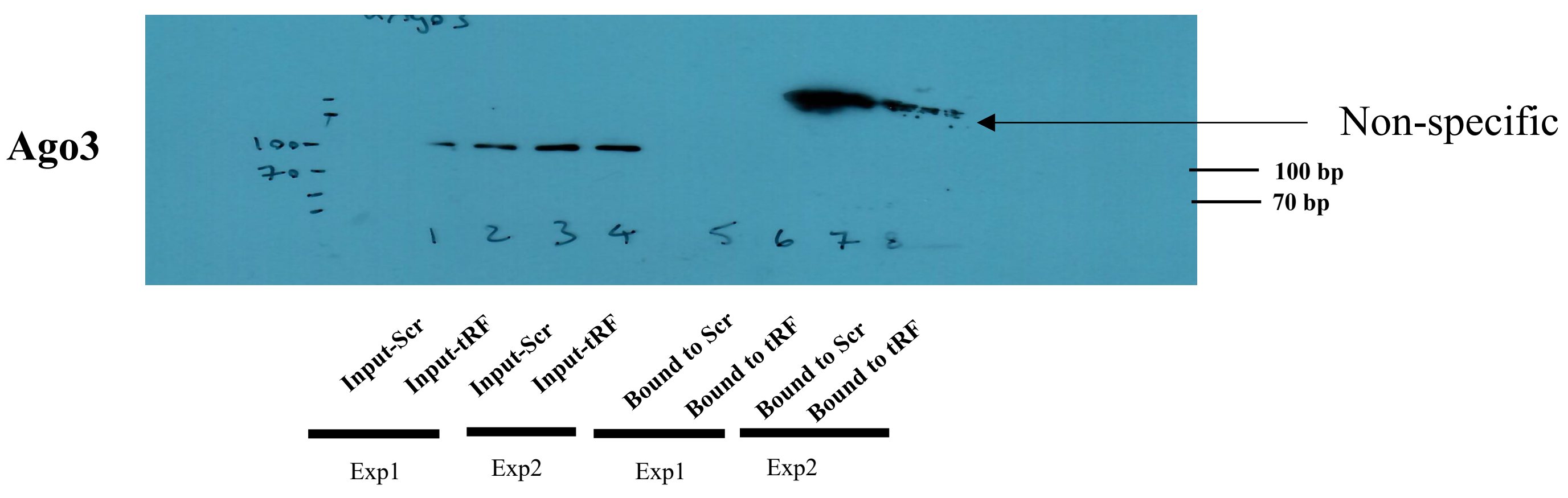

Image captured via ECL and X-ray film
