# Supplementary material for: Leishmania exosomal tRNA-derived small non-coding RNA fragments modulate host THP-1 derived macrophage proteins: A quantitative proteomic analysis
Source: PLoS One. 2026 Aug 19;21(8):e0356144. doi: 10.1371/journal.pone.0356144 (PMC13489484; doi:10.1371/journal.pone.0356144)

S2 Fig: Predicted interactions between the *Leishmania* tRFs and their target genes identified using RNAhybrid with target binding coordinates indicated

1. tRF-Leucine

1. CPT1A (binding region - 5'UTR)

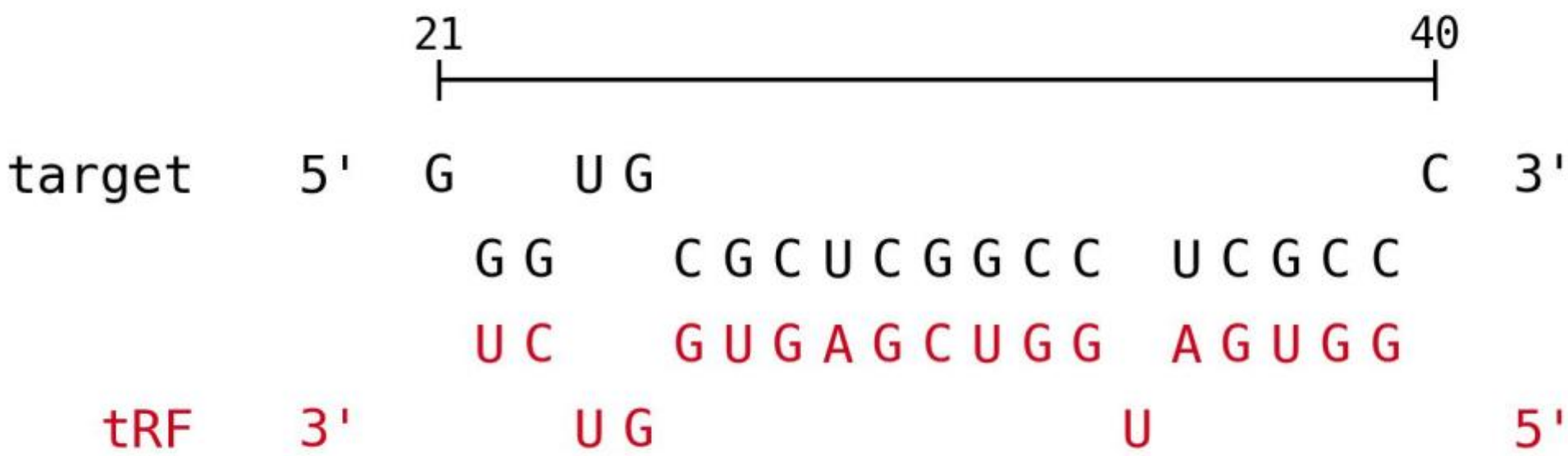

2. SERPINB1 (binding region - 5'UTR)

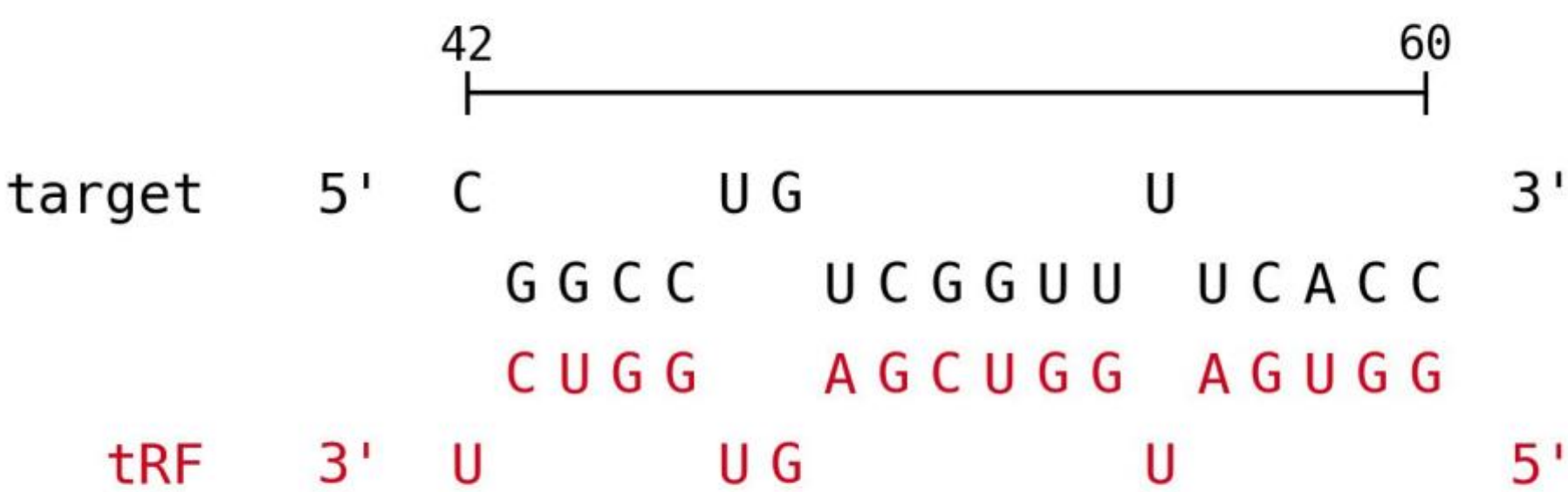

3. LGALS3 (binding region – 5' UTR)

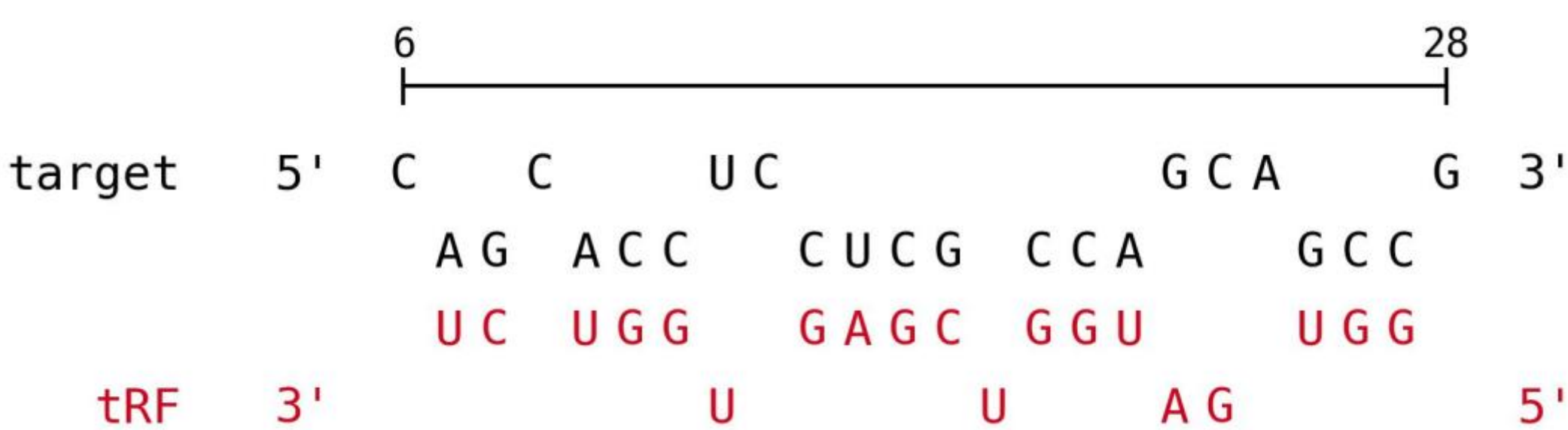

4. TOP2B (binding region – 5' UTR)

```
          346                                  364
          |------------------------------------|
target 5'   C                           C         G  3'
              G C C G C U C G G C C G   C G C C
              U G G U G A G C U G G U   G U G G
   tRF 3' U C                           A            5'
```

5. ATP6V1B2 (binding region – 5' UTR)

```
          1                             16
          |------------------------------|
target 5'             G         G A     G            3'
          G G C C A   U C G G     C A
          U C U G G U   A G C U     G U
   tRF 3'             G         G       A G U G G    5'
```

6. FUNDC2 (binding region – 5' UTR)

```
          56                                     75
          |---------------------------------------|
target 5' C       G       C           C           U  3'
            G G C   C G C   C G G C C   U C   C C
            C U G   G U G   G C U G G   A G   G G
   tRF 3' U               A           U     U        5'
```

2. tRF-Asp

1. AP1B1 (binding region – 5' UTR)

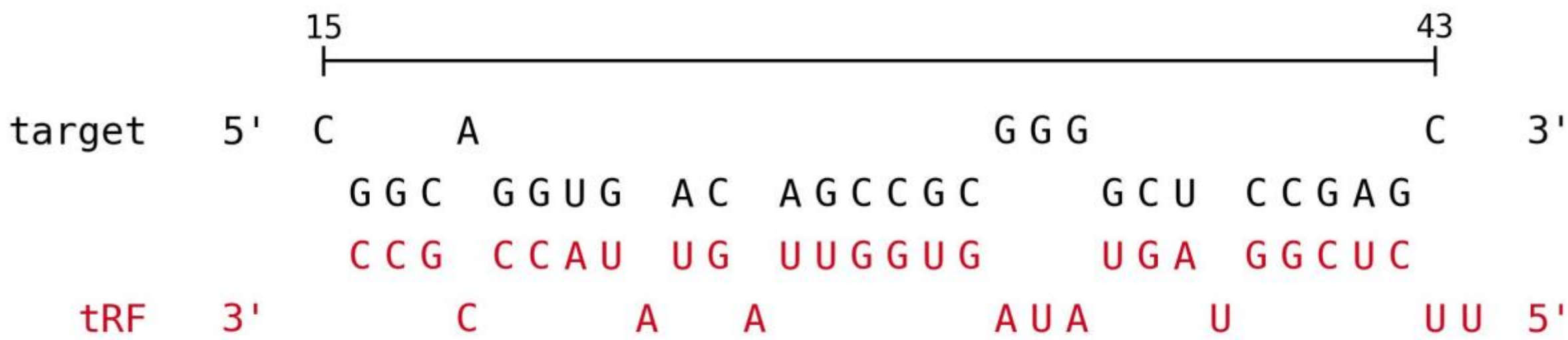

2. ARHGDIA (binding region – 5' UTR)

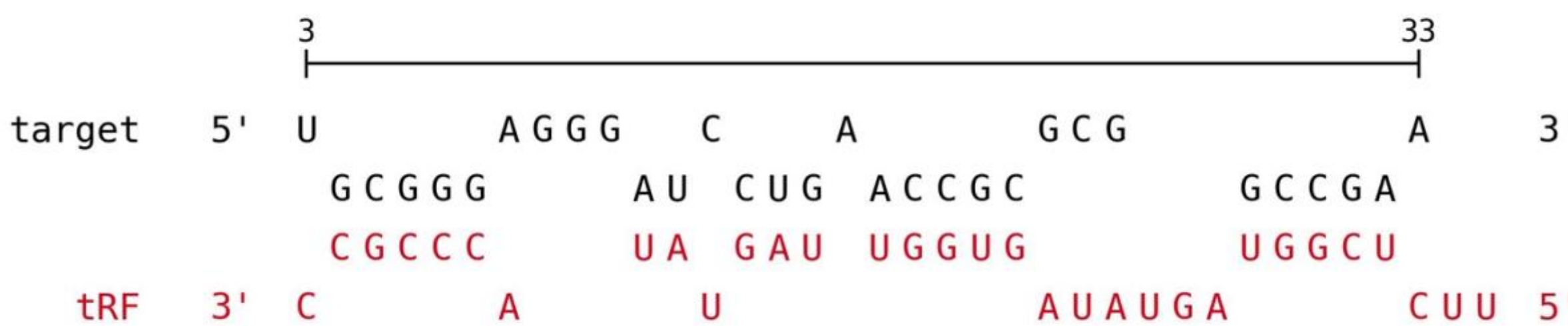

3. H2AZ2 (binding region – CDS)

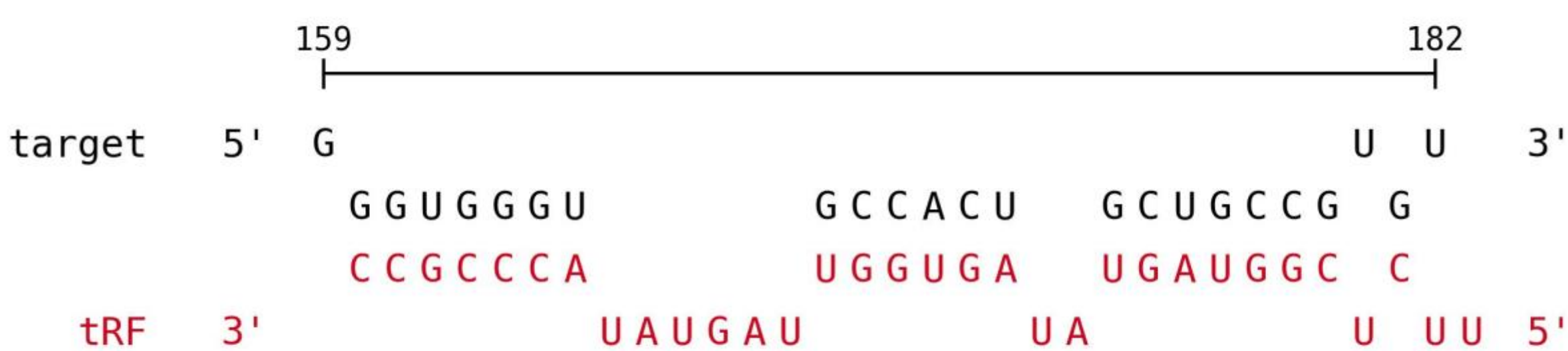

4. STXBP3 (binding region – 5’ UTR)

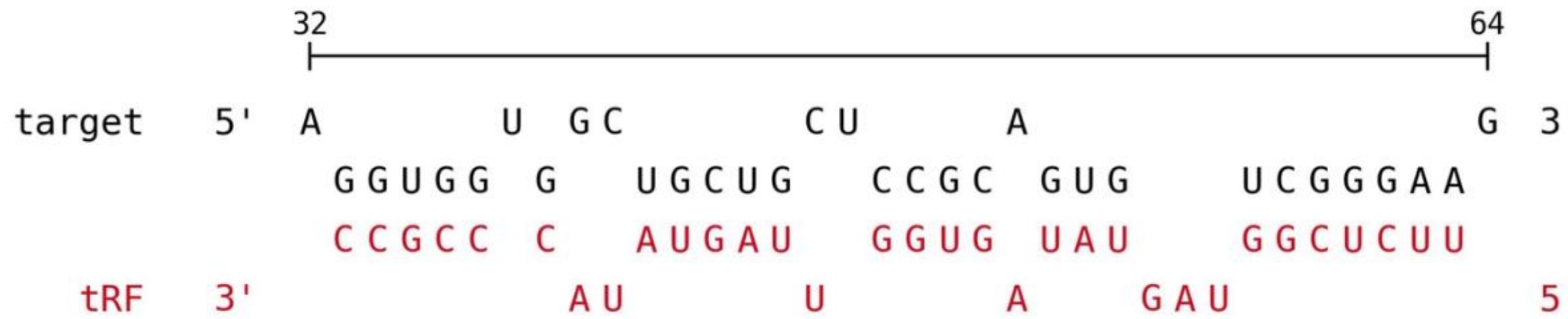

Supplement: Supplementary file 2 [file pone.0356144.s005.pdf]
