# Supplementary material for: Leishmania exosomal tRNA-derived small non-coding RNA fragments modulate host THP-1 derived macrophage proteins: A quantitative proteomic analysis
Source: PLoS One. 2026 Aug 19;21(8):e0356144. doi: 10.1371/journal.pone.0356144 (PMC13489484; doi:10.1371/journal.pone.0356144)
Supplement: Supplementary file 3 [file pone.0356144.s004.docx]

**Table S2A: Sequence of Oligos used for biotin-RNA pull down assays**

| **RNA** | **Sequence** | **Length (nt)** |
| --- | --- | --- |
| ***tRNA-leu-5’*** | ***GGTGAGATGGTCGAGTGGTCT*** | **21** |
| ***Modified sequence,***  ***IDT oligo*** | ***/5BiosG/mGmGmUrGrArGrArUrGrGrUrCrGrArGrUrGrGmUmCmU*** |  |
| ***tRNA- Scrabble-leu-5’*** | ***AGGTTGCGATGGACGGTTGTG*** | **21** |
| ***Modified sequence,***  ***IDT oligo*** | ***/5BiosG/mAmGmGrUrUrGrCrGrArUrGrGrArCrGrGrUrUmGmUmG*** |  |

**Table S2B: Significant predicted interactions between the *Leishmania* tRFs and target genes identified using RNAhybrid.**

Target gene sequences corresponding to the 5′ untranslated region (5′UTR), coding sequence (CDS), and 3′ untranslated region (3′UTR) were retrieved separately from the NCBI Nucleotide database, with MANE Select transcripts preferentially used as canonical isoforms. Minimum free energy (MFE) hybridization between tRF-Leu and target regions was predicted using RNAhybrid (v2.1.2; Rehmsmeier et al., 2004), installed locally through Bioconda within an Anaconda/Python environment. Unless otherwise specified, default RNAhybrid parameters were used. RNAhybrid was run with a maximum target length of 100,000 nt (-m 100000), maximum query length of 50 nt (-n 50), and reporting of the top five predicted binding sites per target (-b 5). Statistical significance was assessed using the human 3′UTR background model (-s 3utr_human). No energy cutoff parameter was applied during prediction, allowing all interactions to be retained for downstream filtering. Predicted binding sites with p < 0.05 and MFE < −20 kcal/mol were considered significant and thermodynamically stable candidate interactions.

| ***tRF-Asp: 5'- TTCTCGGTAGTATAGTGGTTAGTATACCCGCC-3’(33 nt)*** | | | | |
| --- | --- | --- | --- | --- |
| ***Gene*** | ***Region*** | ***MFE (kcal/mol)*** | ***p-value*** | ***Position*** |
| AP1B1 | 5'UTR | -35.6 | 0.003 | 15 |
| ARHGDIA | 5'UTR | -30.6 | 0.005 | 3 |
| H2AZ2 | CDS | -36.2 | 0.011 | 159 |
| STXBP3 | 5'UTR | -31.8 | 0.003 | 32 |
| ***tRF-Leu: 5'- GGTGAGATGGTCGAGTGGTCT -3' (21 nt)*** | | | | |
| ***Gene*** | ***Region*** | ***MFE (kcal/mol)*** | ***p-value*** | ***Position*** |
| CPT1A | 5'UTR | -33.7 | 0.0002 | 21 |
| SERPINB1 | 5'UTR | -26.1 | 0.002 | 42 |
| LGALS3 | 5'UTR | -23.3 | 0.011 | 6 |
| TOP2B | 5'UTR | -33.5 | 0.004 | 346 |
| ATP6V1B2 | 5'UTR | -19.8 | 0.025 | 1 |
| FUNDC2 | 5'UTR | -23.5 | 0.033 | 56 |
